# Human Pathogen Colonization of Lettuce Dependent Upon Plant Genotype and Defense Response Activation

**DOI:** 10.3389/fpls.2019.01769

**Published:** 2020-01-30

**Authors:** Cristián Jacob, Maeli Melotto

**Affiliations:** ^1^ Department of Plant Sciences, University of California, Davis, Davis, CA, United States; ^2^ Horticulture and Agronomy Graduate Group, University of California, Davis, Davis, CA, United States

**Keywords:** lettuce genotypes, fresh produce, genetic diversity, bacterial persistence, human pathogen, *Escherichia coli*, *Salmonella enterica*, disease outbreak

## Abstract

Fresh produce contaminated with human pathogens may result in foodborne disease outbreaks that cause a significant number of illnesses, hospitalizations, and death episodes affecting both public health and the agribusiness every year. The ability of these pathogens to survive throughout the food production chain is remarkable. Using a genetic approach, we observed that leaf colonization by *Salmonella enterica* serovar Typhimurium 14028s (*S.* Typhimurium 14028s) and *Escherichia coli* O157:H7 was significantly affected by genetic diversity of lettuce (*Lactuca sativa* L. and *L. serriola* L.). In particular, there was a significant variation among 11 lettuce genotypes in bacterial attachment, internalization, and apoplastic persistence after surface- and syringe-inoculation methods. We observed a significant correlation of the bacterial leaf internalization rate with stomatal pore traits (width and area). Moreover, bacterial apoplastic populations significantly decreased in 9 out of 11 lettuce genotypes after 10 days of surface inoculation. However, after syringe infiltration, populations of *E. coli* O157:H7 and *S.* Typhimurium 14028s showed positive, neutral, or negative net growth in a 10-day experimental period among seedlings of different lettuce types. The relative ability of the bacteria to persist in the apoplast of lettuce genotypes after syringe inoculation was minimally altered when assessed during a longer period (20 days) using 3.5- to 4-week-old plants. Interestingly, contrasting bacterial persistence in the lettuce genotypes Red Tide and Lollo Rossa was positively correlated with significant differences in the level of reactive oxygen species burst and callose deposition against *S.* Typhimurium 14028s and *E. coli* O157:H7 which are related to plant defense responses. Overall, we characterized the genetic diversity in the interaction between lettuce genotypes and enterobacteria *S.* Typhimurium 14028s and *E. coli* O157:H7 and discovered that this genetic diversity is linked to variations in plant immune responses towards these bacteria. These results provide opportunities to capitalize on plant genetics to reduce pathogen contamination of leaves.

## Introduction

During the last two decades, the number, severity, and distribution of outbreaks of human diseases linked to the consumption of fresh produce have attracted the attention of farmers, the food industry, consumers, politicians, and scientists. According to data reported to the U.S. Centers for Disease Control and Prevention’s Foodborne Disease Outbreak Surveillance System from 1998 and 2013, there were 972 raw produce-associated outbreaks reported, which accounted for 34,674 illnesses, 2,315 hospitalizations, and 72 deaths in the U.S. (Bennett et al., 2018). The most common etiologic agents identified were norovirus (54% of outbreaks), *Salmonella enterica* (21%), and Shiga toxin-producing *Escherichia coli* (10%) (Bennett et al., 2018). This is concerning considering the current upward trend and the steady promotion of fresh produce consumption. In the case of lettuce (*Lactuca sativa* L.), the major ingredient of leafy salads, the U.S. per capita consumption is relatively high at an average of 12.0 kg per person per year in the last decade (ERS-USDA (Economic Research Service - United States Department of Agriculture), 2018). Moreover, the yearly sales of bagged salads have been growing in the U.S., reaching $3.7 billion in 2015 (Cook, 2016), which represents an important change in the consumers’ behavior towards purchasing ready-to-eat and/or minimally processed salads.

Fresh produce is susceptible to contamination by human pathogens from diverse sources during field production, storage, transport, packaging, and processing (Barak and Schroeder, 2012; Sapers and Doyle, 2014). During vegetable production, the major vehicles for bacterial contamination are irrigation water, manure soil amendments, and wild animal intrusion (Jay-Russell, 2013; Allende and Monaghan, 2015; Jiang et al., 2015). For successful phyllosphere colonization, bacteria require the ability to attach, form aggregates, and/or produce biofilms to survive epiphytically. Both *Salmonella* and *E. coli* are able to modulate their metabolism upon leaf contact towards the production of molecules involved in attachment and biofilm matrix formation (Yaron and Römling, 2014). Phylloplane settlement processes are followed and/or accompanied by the bacterial movement toward and through the stomatal pore. Studies have demonstrated that both *Salmonella* and *E. coli* are able to reach the leaf intercellular space through the stomatal pore (Seo and Frank, 1999; Kroupitski et al., 2009; Saldaña et al., 2011; Roy et al., 2013). Plant cell recognition of Microbe-Associated Molecular Patterns (MAMPs) of human bacterial pathogens can trigger the production of Pattern-Triggered Immunity (PTI)-associated defense responses (Garcia et al., 2014), including a decrease in stomatal aperture width (Melotto et al., 2006; Roy et al., 2013). On the other hand, bacteria might counter-attack the plant responses by subverting the stomatal closure defense (Roy et al., 2013) or activating genes associated with oxidative stress tolerance and antimicrobial resistance (Van der Linden et al., 2016).

The overall outcome of the interaction between plants and human bacterial pathogens on/in the leaf is the persistence of the microorganisms for few days to several weeks (Solomon et al., 2003; Islam et al., 2004a; Islam et al., 2004b; Fonseca et al., 2011; Kisluk and Yaron, 2012). The ability of bacteria to survive in the phyllosphere is largely dependent upon the plant species and specific genotypes of each species (Klerks et al., 2007; Mitra et al., 2009; Barak et al., 2011; Golberg et al., 2011; Quilliam et al., 2012; Macarisin et al., 2013; Hunter et al., 2015; Crozier et al., 2016; Erickson et al., 2018; Roy and Melotto, 2019). Certain leaf traits have been associated with intraspecific and interspecific differences in plant colonization, together with variation between and within plant tissues. For instance, Macarisin et al. (2013) found differential persistence of *E. coli* O157:H7 on the leaves of spinach cultivars, which was influenced by leaf blade roughness and stomatal density. Other leaf surface factors, such as vein density, hydrophobicity, and level of epicuticular wax, were associated with cultivar-specific differences in *S. enterica* ser. Senftenberg attachment on iceberg and Batavia type lettuces (Hunter et al., 2015). In tomato, the level of *S. enterica* persistence in the phyllosphere after dip-inoculation with an eight-serovar cocktail (serovars Baildon, Cubana, Enteritidis, Havana, Mbandaka, Newport, Poona, and Schwarzengrund) also seems to be influenced by plant genotype (Barak et al., 2011). Furthermore, *S. enterica* seedling colonization of lettuce and tomato has been reported not only to be influenced by the plant species and cultivar, but also by the bacterial serovar and strain (Wong et al., 2019).

Although there is evidence indicating that plant genotypic diversity influences the colonization of the phyllosphere by human bacterial pathogens, phenotypes associated with the observed differences are limited to the morphological and chemical composition of the leaf surface. Molecular mechanisms and biological processes involved in the variation of bacterial survival in the phyllosphere of different plant genotypes are largely unknown. Moreover, variation in the interaction between plants and human pathogenic bacteria due to plant genetic diversity has been shown to be quantitative (Barak et al., 2011; Quilliam et al., 2012; Marvasi et al., 2014). Therefore, few to several genetic factors might be influencing variation in the resulting phenotype (Corwin and Kliebenstein, 2017). This complex scenario exposes the necessity to find robust phenotypic differences in a phyllosphere-human pathogenic bacterium system that enables an in-depth analysis of the underlying factors. In this study, we characterized the genetic diversity in the interaction between lettuce genotypes and the enterobacteria *S. enterica* Typhimurium 14028s and *E. coli* O157:H7. Furthermore, we discovered that this genetic diversity is linked to differences in the plant immune responses.

## Materials and Methods

### Plant Material and Growth Conditions

A set of 11 lettuce genotypes was used to conduct this study (**Table 1**). Seeds were sown on water-soaked germination paper in square Petri dishes and incubated for 2 days at 20°C. Germinated seeds were transferred to either peat moss pellets (42 mm, Jiffy^®^ 7, Canada) or to 7.62 cm^2^ pots (Kord Products, Toronto, Canada) containing a commercial soil mix (Sun Gro^®^ Sunshine^®^ #1 Grower Mix with RESiLIENCE™, MA, USA). Plants were grown under photosynthetic active light intensity of 240 ± 10 µmol m^-2^ sec^-1^ with a 12-hour photoperiod. Relative humidity (RH) and temperature were recorded every 15 min with a data logger (GSP-6, Elitech^®^, CA, USA). Day and night conditions were 19 ± 1°C and 75 ± 4% RH and 18 ± 1°C and 92 ± 2% RH, respectively. One liter of tap water was added to the tray two to three times per week depending on the developmental stage of the plants. At 10 days after germination, 0.05 g/plant of fertilizer (Multi-Purpose 19-11-21, Peters^®^Excel, OH, USA) was dissolved in the irrigation water.

**Table 1:** List of lettuce genotypes used to evaluate the natural genetic variability regarding the plant response to *Salmonella enterica* Typhimurium 14028s and *Escherichia coli* O157:H7.

Species	Genotype/lettuce type	Accession number	Life cycle^1^	Plant disease traits			
				Resistance		Susceptibility	
				*Pathogen*	*Reference(s)*	*Pathogen*	*Reference(s)*
*Lactuca sativa* L. var. *crispa* L.	Salinas/Iceberg	14G1846-1	Long	Dieback (caused by two viruses from the family Tombusviridae)	Grube and Ochoa (2005); Simko et al. (2009)	Lettuce big-vein associated virus	Ryder and Robinson (1995)
				*Fusarium oxysporum* f.sp. *lactucae* race 1	McCreight et al. (2005)	*Sclerotinia minor*	Hayes et al. (2010)
				*Fusarium oxysporum* f.sp. *lactucae*	Scott et al. (2010)	*Verticillium dahliae*	Vallad and Subbarao (2008)
						*Bremia lactucae*	Grube and Ochoa (2005); Simko et al. (2014)
*L. sativa* L. var. *crispa* L.	Emperor/Iceberg	14G11-1	Long				
*L. sativa* L. var. *crispa* L.	La Brillante/Batavia	13G637-2	Short	Dieback	Simko et al. (2009)	.	
				Lettuce big-vein associated virus	Ryder and Robinson (1995)		
				*Bremia lactucae*	Simko et al. (2015)		
				*Xanthomonas campestris* pv. *vitians*	Hayes et al. (2014)		
				*Verticillium dahliae*	Vallad and Subbarao (2008)		
*L. sativa* L. var. *acephala* Dill.	Lollo Rossa/Red loose leaf	10G11-2	Short	*Xanthomonas campestris* pv. *vitians*	Hayes et al. (2014)	Dieback	Simko et al. (2009)
				*Fusarium oxysporum* f.sp. *lactucae*	Scott et al. (2010)		
*L. sativa* L. var. *acephala* Dill.	Red Tide/Red loose leaf	10G12-2	Short	*Sclerotinia sclerotiorum*	Hayes et al. (2010)	Dieback	Simko et al. (2009)
						*Bremia lactucae*	Simko et al. (2014)
*L. sativa* L. var. *acephala* Dill.	Grand Rapids/Green loose leaf	13G1033-1	Short	Dieback	Grube et al. (2003)		
				*Bremia lactucae*	Grube and Ochoa (2005)		
*L. sativa* L. var. *longifolia* (Lam.) Janchen	Green Towers/Romaine	14G388-2	Medium	*Sclerotinia sclerotiorum*	Hayes et al. (2010)	Dieback	Grube et al. (2003); Simko et al. (2009)
						*Bremia lactucae*	Grube and Ochoa (2005)
						*Phoma exigua*	Grube et al. (2003)
*L. sativa* L. var. *capitata* (L.) Janchen	Bibb/Butterhead	15G6-1	Medium	Dieback	Simko et al. (2009)	*Sclerotinia sclerotiorum*	Whipps et al. (2002)
*L. sativa* L.	PI 251246/Oilseed	13G640-1	Very short	Dieback	Simko et al. (2009)		
				*Sclerotinia sclerotiorum*	Whipps et al. (2002); Hayes et al. (2010)		
*L. serriola* L.	Serriola I/Prickly lettuce	12G239-1	Short				
*L. serriola* L.	Serriola II/Prickly lettuce	UC23US96	Short			*Bremia lactucae*	Simko et al. (2014)

^1^Relative differences in life cycle (from seed to seed) under the same environmental conditions. Long = 5–6 months, Medium = 4–5 months, Short = 3–4 months, and Very Short <3 months. Note that all *L. sativa* genotypes, except PI 251246/Oilseed, are commercial cultivars of lettuce.

### Bacterial Strains and Preparation of Inoculum

The non-typhoid *S. enterica* subspecies *enterica* serovar Typhimurium strain 14028s (Porwollik et al., 2014) (hereafter *S.* Typhimurium 14028s) and the enterohemorrhagic *E. coli* serotype O157:H7 strain 86-24 (Sperandio et al., 2001) (hereafter *E. coli* O157:H7) were grown in Low Salt Luria-Bertani (LSLB) medium (10 g/L tryptone, 5 g/L yeast extract, 5 g/L NaCl, pH 7.0) at 28°C. Medium supplemented with 50 µg/mL of streptomycin was used to grow *E. coli* O157:H7. Bacterial culture for the preparation of inoculum was obtained by streaking cells from frozen glycerol stocks on to solid LSLB medium and incubating overnight. From this culture, a single colony was used to inoculate liquid LSLB medium, which was incubated until reaching an OD_600_ of 0.9 to 1. Bacterial cells were collected by centrifugation at 1,360 x *g* for 20 min at 20°C (Eppendorf Centrifuge 5810R, Rotor 157 A-4-81, Hamburg, Germany) and suspended in sterile distilled water (SDW) to obtain the desired inoculum concentration.

### Bacterial Attachment Assay

Bacterial attachment to the lettuce leaf surface was assessed as described by Van der Linden et al. (2014) with some modifications. Specifically, the inoculum was prepared in 1 mM MgCl_2_ to avoid bacterial osmotic stress during the incubation period and the bacterial concentration in the inoculum was 1 x 10^8^ CFU/mL. For sampling consistency, the second, fully expanded leaf of three 2.5- to 3-week-old plants grown in peat moss pellets were excised from the base of the petiole. Each leaf was immersed in an open 50 mL tube containing 45 mL of inoculum, preventing contact between the cut zone and the inoculum. Tubes were incubated for 2 hours without agitation at room temperature and a photosynthetic active light intensity of 240 ± 10 µmol m^-2^ sec^-1^. After incubation, leaves were rinsed twice in SDW for 1 min and then blotted on a paper towel. Then, the bacterial population was enumerated by serial-dilution plating as described by Jacob et al. (2017). Three leaves from each genotype were used for each treatment and the experiment was repeated four times (n = 12) with independent batches of plants.

### Analysis of Leaf Surface Traits

Leaf surface traits were quantified using the Nikon Eclipse 80i fluorescent microscope and the NIS Elements Imaging Software version 4.13.04 (Nikon Corporations, Shinagawaku, Tokyo, Japan). All measurements were conducted on the abaxial side of the second leaf from three 2.5- to 3-week-old plants grown in peat moss pellets. Leaf pieces (~0.5 cm^2^) were cut from each side of the midrib (six pieces of leaf per genotype) and immediately imaged under the microscope. Stomatal pore traits (aperture width and pore area) were quantified in 18 randomly chosen pictures taken at 6 hours after first light (n = 45 to 60 stomata). To calculate the stomatal density (number of stomata per mm^2^ of leaf), four randomly chosen microscopic fields of view from each piece of leaf (n = 24) were used. All quantifications were carried out three times using independently grown batches of plants.

### Leaf Surface Inoculation

Surface-inoculation was conducted to evaluate the bacterial internalization rate (IR) and subsequent bacterial survival in the leaf intercellular space of lettuce genotypes. The protocol was adapted from those previously described for the pathosystem *Arabidopsis thaliana*–*Pseudomonas syringae* (Katagiri et al., 2002; Jacob et al., 2017). Lettuce plants (2.5- to 3-week-old) grown in peat moss pellets were used. Surface inoculation consisted of dipping for 5 seconds the aerial part of the plants in 200 mL of inoculum (1 x 10^8^ CFU/mL) containing 0.03% Silwet L-77 (Lehle Seeds Co., Round Rock, TX, USA). The second leaf of each plant was sampled to quantify the bacterial population at 0, 1, 5, and 10 days post inoculation (DPI). Four 0.2 cm^2^ discs, punched with a cork-borer, were placed in a 1.7 mL centrifuge tube and ground in 100 µL of SDW. The bacterial population was enumerated by serial-dilution plating as described by Jacob et al. (2017). To quantify the bacterial IR and subsequent apoplastic persistence, bacterial enumeration on day 0 was conducted in non-surface sterilized leaves; for the rest of the sampling points, leaves were gently washed in 2% (v/v) sodium hypochlorite for 1 min, 70% (v/v) ethanol for 1 min, rinsed in SDW for 1 min, and blotted onto a paper towel. This surface-sterilization method was optimized to kill all *S.* Typhimurium 14028s and *E. coli* O157:H7 on the leaf surface. The IR was estimated as the ratio of CFU/cm^2^ leaf at 1 DPI over that of at 0 DPI. Three leaves from three plants were used for each sample point per genotype and the experiment was conducted three times (n = 9) with independent batches of plants.

### Leaf Apoplast Inoculation

Syringe-infiltration inoculation was conducted to evaluate the bacterial survival in the leaf intercellular space of the lettuce genotypes. Lettuce seedlings (2.5 to 3 weeks old) grown in peat moss pellets were subjected to syringe infiltration and sampled at 0 and 10 DPI; similarly, 3.5- to 4-week-old plants grown in pots were subjected to syringe infiltration and sampled at 0, 10, and 20 DPI. The inoculum (1 x 10^6^ CFU/mL) was infiltrated into the apoplastic space using a needleless syringe according to Katagiri et al. (2002). The bacterial population was enumerated by serial-dilution plating as described by Jacob et al. (2017). Leaves were surface sterilized prior to bacteria enumeration by gently washing in 2% (v/v) sodium hypochlorite for 1 min, 70% (v/v) ethanol for 1 min, rinsed in SDW for 1 min, and blotted onto a paper towel. Three leaves were used for each sample point per genotype and the experiment was conducted three times (n = 9) with independent batches of plants.

### ROS Burst Assay

Apoplastic reactive oxygen species (ROS) were quantified through a fast and robust bioassay, as described by Smith and Heese (2014). Leaf discs (0.2 cm^2^) from the second leaf of 2.5- to 3-week-old plants were placed individually into wells of a 96-well microplate containing 200 µL of SDW and incubated overnight at constant light and 22°C to reduce the wounding response. After incubation, SDW was replaced with 100 µL of the elicitation solution composed of 5.38 units of Horseradish Peroxidase (MilliporeSigma, Burlington, MA, USA) and 34 µg of Luminol (MilliporeSigma, Burlington, MA, USA) per mL of SDW with or without 5 x 10^8^ CFU/mL of *E. coli* O157:H7 or *S.* Typhimurium 14028s. The elicitation solution containing bacteria was prepared with heat-killed bacterial suspensions (incubated at 100°C for 10 minutes) to avoid possible inhibition of ROS production by any unknown virulence factor produced by live bacteria in contact with leaf tissue. After adding the elicitation solution to the wells, plates were immediately inserted in a microplate reader (Synergy H1 Hybrid Multi-Mode Reader, Biotek, Winooski, VT, USA) to measure luminescence and estimate ROS production every 2 minutes between 0 and 90 minutes. For each treatment, 24 leaf discs were collected from six different plants. The experiment was repeated five times with independent batches of plants.

### Callose Deposition Assay

A callose deposition assay was performed according to the procedure described by Schenk and Schikora (2015). The second leaf of 2.5- to 3-week-old plants was syringe-infiltrated with either water (mock treatment) or 1 x 10^8^ CFU/mL of bacterium inoculum. After 24 hours, leaves were harvested, and chlorophyll was cleared by immersing the leaves into 95% (v/v) ethanol and kept at 37°C for 24 hours in a rotary shaker. Cleared leaves were rinsed in 50% (v/v) ethanol for 1 min, SDW for 1 min twice, 50 mM K_2_HPO_4_ for 3 min, followed by a 1 hour incubation in a 150 mM K_2_HPO_4_ SDW based solution containing 0.05% aniline blue. Leaves were imaged with a Nikon Eclipse 80i fluorescent microscope (Nikon Corporations, Shinagawaku, Tokyo, Japan) equipped with a DAPI (4’,6-diamidino-2-phenylindole) filter, and the NIS Elements Imaging Software Version 4.13.04 was used to process images. Three leaves of each genotype were used per treatment and six images were randomly captured from each side of the midrib (12 pictures per leaf). Infiltrated zones, damaged areas, mid vein, and leaf edges were avoided for imaging to prevent false positive results. The total area of callose deposits (mm^2^ per cm^2^ of leaf) was quantified using the binary tool of the abovementioned software. The experiment was repeated four times with independent batches of plants.

### Statistical Analysis

To assess the effect of lettuce genotype and bacterium species on bacterial leaf attachment, internalization rate, and apoplastic persistence after surface inoculation, the data was subjected to a two-way analysis of variance (ANOVA) followed by comparisons of multiple means using Tukey’s test with a significance threshold of α = 0.05. The statistical analysis of the bacterial persistence was conducted with the data transformed with the square root function as recommended for data where the variance is proportional to the mean, as often happens in variables that are measured as counts per area (Manikandan, 2010). However, the graphs showing bacterial enumeration were created with untransformed values. Averages of bacterial population after syringe inoculation (10 or 20 DPI versus 0 DPI) and averages of plant defense responses (Lollo Rossa versus Red Tide) were compared using a two-tailed Student’s *t*-test. To evaluate the strength of the linear correlation between quantitative variables, bacterial leaf colonization traits and stomatal traits, a Pearson’s correlation test was conducted. Statistical analysis was done using InfoStat/E software version 2016e (Agricultural College of the National University of Córdoba, Argentina) and R software version 3.5.1 (R Foundation for Statistical Computing, Vienna, Austria. https://www.r-project.org/).

## Results

### Attachment of *S.* Typhimurium 14028s and *E. coli* O157:H7 Onto Lettuce Leaves

**Figure 1:**
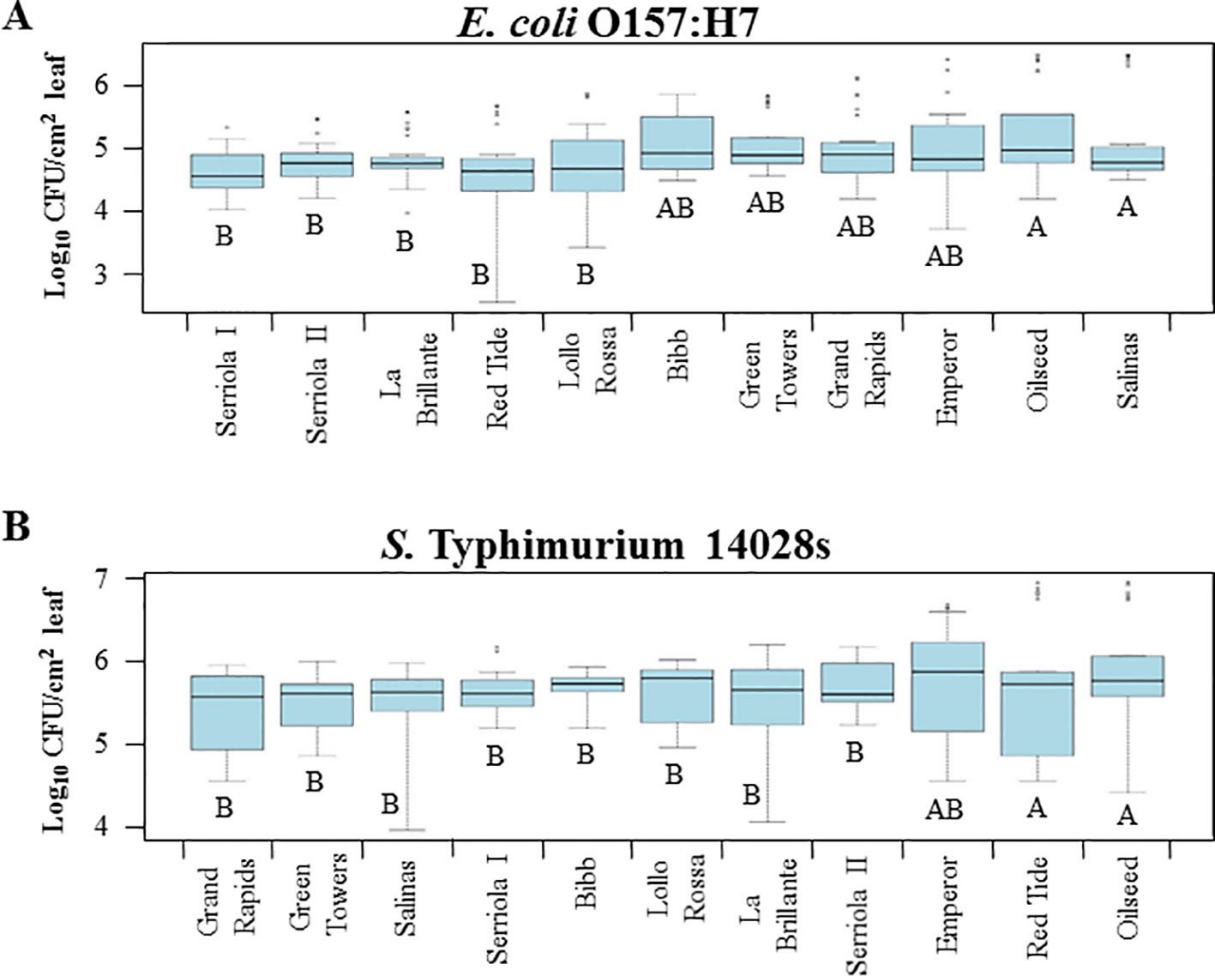
*Escherichia coli* O157:H7 **(A)** and *Salmonella enterica* Typhimurium 14028s **(B)** attachment to the leaf surface varies among lettuce genotypes. The second leaf of 2.5- to 3-week-old lettuce plants was immersed in 1 x 10^8^ CFU/mL bacterial inoculum for 2 hours at room temperature. After incubation, leaves were rinsed with sterile distilled water and bacterial population was enumerated by serial dilution plating. Plots show data from four independent experiments (n = 12). Different letters on the bottom of the boxes indicate significant statistical differences among the means, as calculated with ANOVA followed by Tukey’s test (α = 0.05).

Bacterial attachment to leaf surface is largely affected by various topographic traits (Crawford et al., 2012). In lettuce, leaf vein and stomatal densities, leaf surface hydrophobicity, soluble protein concentrations, and wax content have been reported as factors influencing differences in the attachment of *S. enterica* ser. Senftenberg among lettuce genotypes and leaves at different developmental stages (Hunter et al., 2015). Our results show significant variation (p < 0.0001) in the attachment of *E. coli* O157:H7 and *S.* Typhimurium 14028s among lettuce genotypes (**Figure 1**). Attachment of *E. coli* O157:H7 ranged from 5.4 x 10^4^ ± 8.3 x 10^3^ (mean ± standard error) to 5.8 x 10^5^ ± 1.9 x 10^5^ CFU/cm^2^ leaf from Serriola I to Salinas, respectively (**Figure 1A**). In contrast, extension of variation of *S.* Typhimurium 14028s leaf surface attachment ranged from 1.9 x 10^6^ ± 5.1 x 10^5^ CFU/cm^2^ on Oilseed leaves to 3.9 x 10^6^ ± 5.1 x 10^5^ CFU/cm^2^ on Grand Rapids leaves (**Figure 1B**).

**Figure 2:**
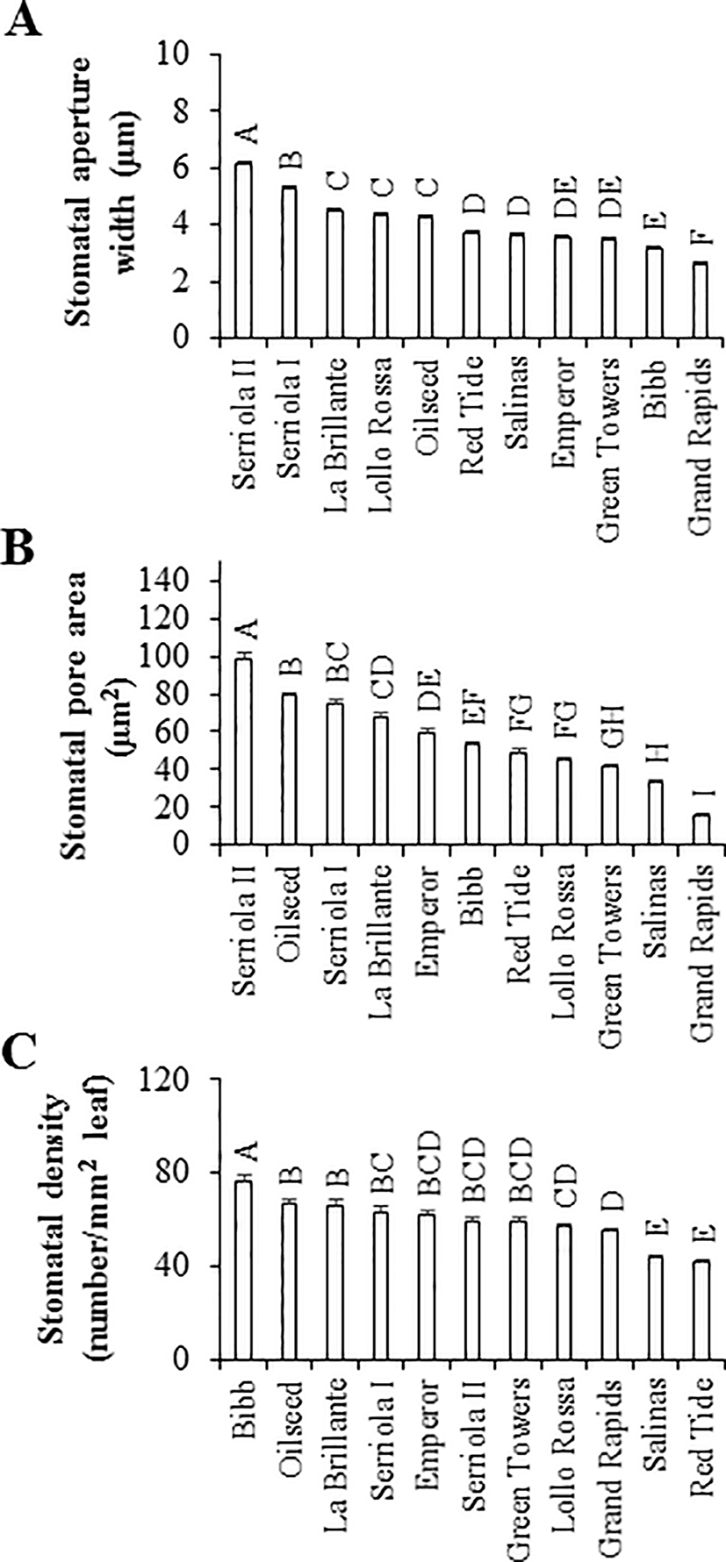
Stomatal characteristics vary among the lettuce genotypes. Stomatal aperture width **(A)** and pore area **(B)** were measured in 45 to 60 stomata at 6 hours after first light. Stomatal density **(C)** was calculated in eight randomly chosen microscopic fields of view from three leaves (n = 24). All measurements were taken from the abaxial side of the second leaf from 2.5- to 3-week-old plants. Stomatal characteristic data are shown as average ± standard error from three experiments using different batches of plants. Different letters on the top of the bars indicate significant statistical differences among the means, as calculated with ANOVA followed by Tukey’s test (α = 0.05).

Considering the significant variation in the bacterial attachment onto leaves, we determined the stomatal density together with the stomatal aperture width and stomatal pore area of the lettuce genotypes to assess the potential correlation between these traits and bacterial attachment. Stomatal characteristics varied significantly (p < 0.0001) among the eleven lettuce genotypes (**Figure 2**). The two *L. serriola* genotypes, Serriola II and Serriola I, had the widest stomatal aperture width, averaging 6.1 ± 0.1 and 5.3 ± 0.1 µm, respectively, while Grand Rapids had the smallest stomatal aperture width (2.6 ± 0.1 µm; **Figure 2A**). Stomatal pore area also varied significantly among lettuce genotypes, from 98.6 ± 3.0 µm^2^ in the genotype Serriola II to 15.6 ± 0.7 µm^2^ in Grand Rapids (**Figure 2B**). Regarding stomatal density, this trait showed genotypic variation ranging from 76.0 ± 2.1 to 41.7 ± 1.0 stomata/mm^2^ in the genotypes Bibb and Red Tide, respectively (**Figure 2C**). These stomatal traits (aperture width, pore area, and density) presented no significant correlation with *E. coli* O157:H7 or *S.* Typhimurium 14028s attachment onto leaves of the different lettuce genotypes (**Table 2**). Thus, these results suggest that bacterial attachment might be influenced by properties of the leaf surface and by specific bacterial traits (*e.g*., motility, chemotaxis) on the phyllosphere.

**Table 2:** Correlation (Pearson’s coefficient and p-value) between *Escherichia coli* O157:H7 or *Salmonella enterica* Typhimurium 14028s leaf colonization traits [leaf attachment, internalization rate (IR), and bacterial population 10 days post surface inoculation (10 DPI)] and stomatal traits (stomatal aperture width, stomatal pore area, and stomatal density) among the 11 lettuce genotypes.

Bacteria	Bacterial colonization trait	Pearson correlation	Stomatal trait		
			Aperture width	Pore area	Density
***E. coli*O157:H7**	**Attachment**	Coefficient (*r*)	-0.41	-0.29	-0.19
		p-value	0.216	0.379	0.575
	**IR**	Coefficient (r)	0.77	0.71	0.36
		p-value	***0.005***	***0.015***	0.274
	**10 DPI**	Coefficient (r)	0.75	0.67	0.19
		p-value	***0.008***	***0.025***	0.570
***S.* Typhimurium 14028s**	**Attachment**	Coefficient (r)	0.06	0.28	-0.22
		p-value	0.860	0.405	0.516
	**IR**	Coefficient (r)	0.72	0.64	0.02
		p-value	***0.012***	***0.032***	0.954
	**10 DPI**	Coefficient (r)	0.48	0.49	-0.13
		p-value	0.139	0.128	0.704

Significant p-values (< 0.05) are in bold italic numbers.

### Internalization Rate of *S.* Typhimurium 14028s and *E. coli* O157:H7

**Figure 3:**
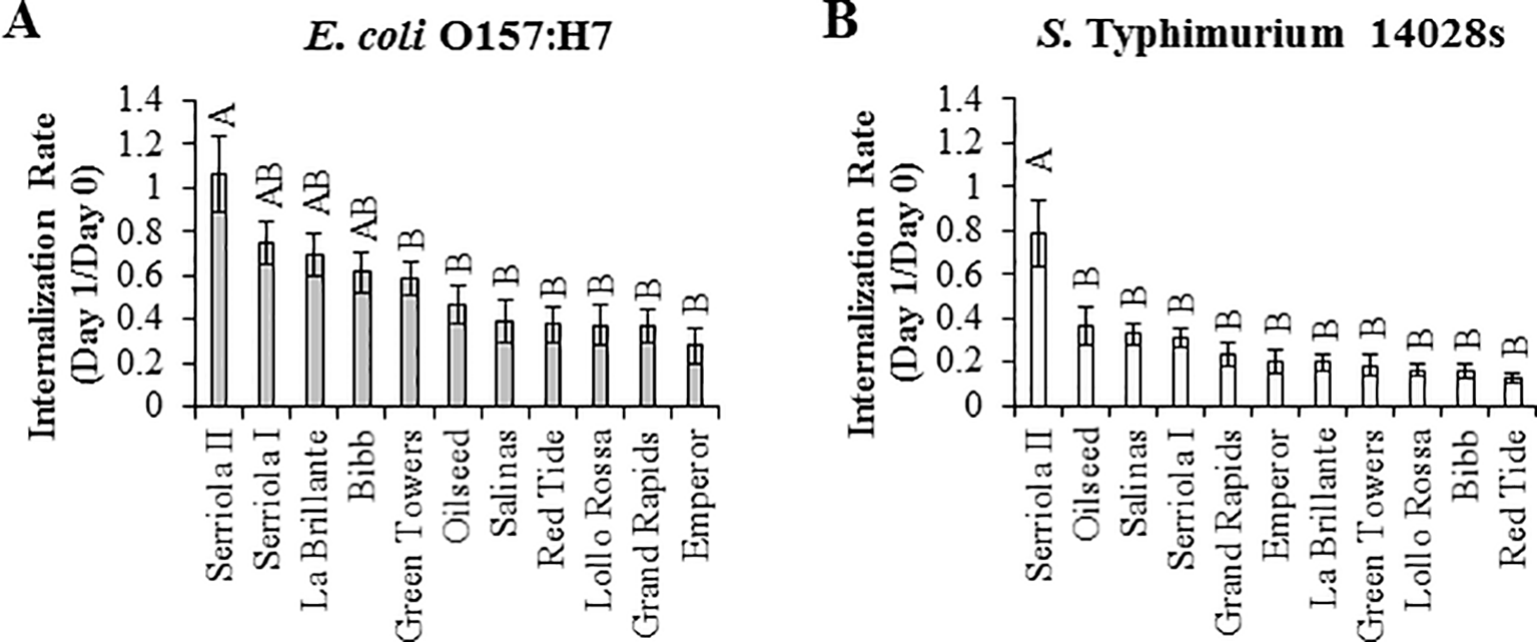
The internalization rate of *Escherichia coli* O157:H7 **(A)** and *Salmonella enterica* Typhimurium 14028s **(B)** vary among the lettuce genotypes. Internalization rate was calculated as the ratio of bacterial concentration (CFU/cm^2^ leaf) between day 1 and day 0 after surface inoculation (1 x 10^8^ CFU/mL). Bacterial population was quantified in intact leaves for day 0 after inoculation and in leaves previously surface sterilized for day 1 after inoculation. Data shown is the average of three independent experiments (n = 9). Different letters on the top of the bars indicate significant statistical differences among the means, as calculated with ANOVA followed by Tukey’s test (α = 0.05).

Previously, we determined that STm SL1344 and *E. coli* O157:H7 penetrate leaves through the stomatal pore (Roy et al., 2013). Here, we observed significant differences (p < 0.0001) in the internalization rate (IR; estimated as the ratio of CFU/cm^2^ leaf at 1 DPI over that of at 0 DPI) of these bacteria among the lettuce genotypes (**Figure 3**). *E. coli* O157:H7 IR varied from 1.07 ± 0.17 in Serriola II to 0.28 ± 0.08 in Emperor, while *S.* Typhimurium 14028s IR ranged from 0.79 ± 0.15 to 0.13 ± 0.02 in Serriola II and Red Tide, respectively (**Figure 3**). Interestingly, we found a significant correlation (p < 0.05) between the IR of both human pathogenic bacteria and stomatal traits (aperture width and pore area) among the eleven lettuce genotypes (**Table 2**). No significant correlation between stomatal density and bacterial IR was detected (**Table 2**). These results suggest that, to a certain extent, morphological traits of the stomatal pore (width and area) contribute to bacterial penetration into the lettuce leaf.

### *E. coli* O157:H7 and *S.* Typhimurium 14028s Persistence After Surface Inoculation

**Figure 4:**
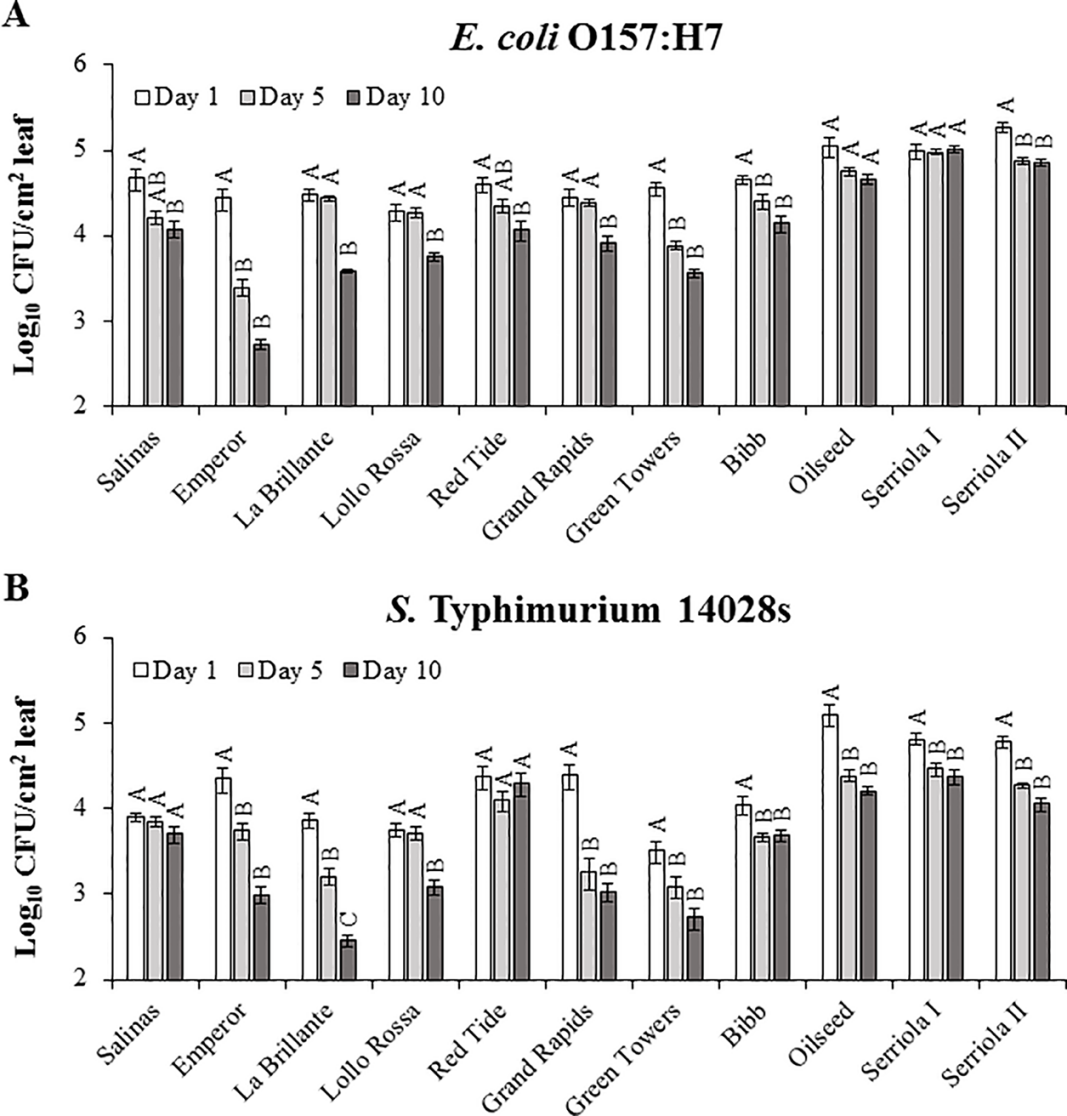
Persistence of *Escherichia coli* O157:H7 **(A)** and *Salmonella enterica* Typhimurium 14028s **(B)** after surface inoculation of leaves varies with lettuce genotypes. Lettuce plants (2.5- to 3-week-old) were dipped into 1 x 10^8^ CFU/mL bacterial inoculum. Leaves were surface sterilized prior to quantification of the bacterial population in the intercellular space. Results are shown as untransformed averages from three independent experiments (n = 9 ± standard error). Different letters on the top of adjacent bars (*i.e.*, within the plant genotype) indicate significant statistical differences among the means (transformed with the root square function), as calculated with ANOVA followed by Tukey’s test (α = 0.05).

Previous studies have demonstrated that survival of *E. coli* O157:H7 and different *S. enterica* serovars in/on leaves is significantly affected by the plant genotype, such as in spinach and tomato (Mitra et al., 2009; Barak et al., 2011; Gutiérrez-Rodríguez et al., 2011; Macarisin et al., 2013; Han and Micallef, 2014). Thus, we sought to determine whether this phenomenon is also true for the lettuce system. Similarly, we observed that changes in internalized *E. coli* O157:H7 and *S.* Typhimurium 14028s populations throughout the experimental period (10 DPI) were significantly (p < 0.0001) influenced by lettuce genotype and the species of bacteria. With the exception of a few genotypes, bacterial populations in the apoplast decreased significantly from 1 DPI to 10 DPI in the different plant-bacterium combinations (**Figure 4**). The average log change in the *E. coli* O157:H7 population varied from a non-significant 0.03 log increment in Serriola I to a significant 1.71 log reduction in Emperor (**Figure 4A**), while the *S.* Typhimurium 14028s population change ranged from a non-significant 0.09 log reduction in Red Tide to a significant 1.41 log reduction in La Brillante (**Figure 4B**). Although the population of both inoculated bacteria generally decreased with the duration of the experiment, the kinetics and the extent of the decrease were significantly different (p < 0.0001) among the lettuce genotypes. It is noteworthy that enumerating internalized population size after surface inoculation represents a combined effect of leaf surface features, bacterium IR, and endophytic persistence. The bacterial population at the end of the experimental period (10 DPI) exhibited a significant correlation (p < 0.05) with stomatal aperture width and pore area for *E. coli* O157:H7 but not for *S.* Typhimurium 14028s (**Table 2**). Therefore, these findings suggest that each lettuce genotype may have a combination of features both on the leaf surface and in the intercellular space, which hamper or facilitate the persistence of these two human pathogens.

### *S.* Typhimurium 14028s and *E. coli* O157:H7 Persistence in 2.5- to 3-Week-Old Plants After Inoculum Infiltration

**Figure 5:**
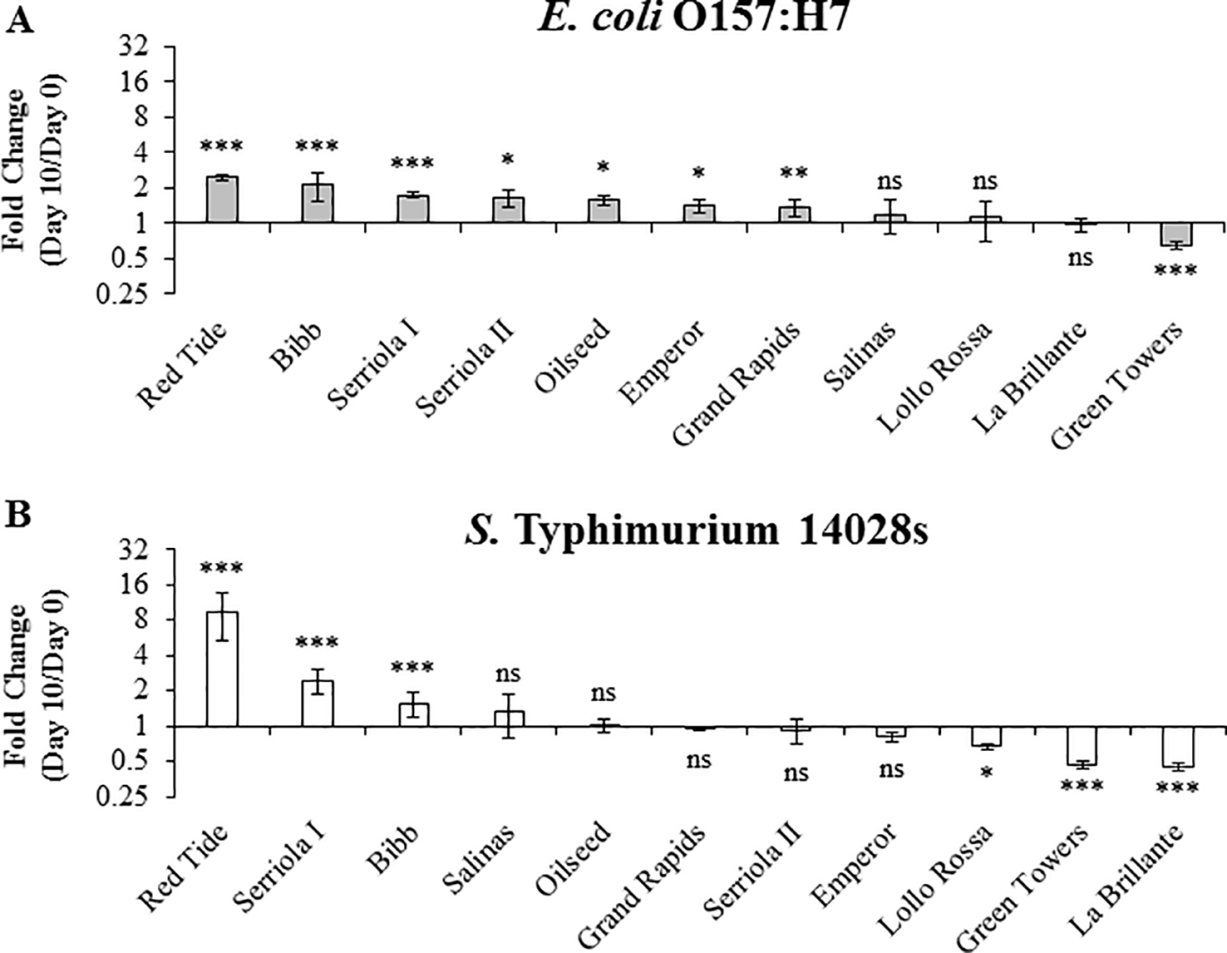
Persistence of *Escherichia coli* O157:H7 **(A)** and *Salmonella enterica* Typhimurium 14028s **(B)** varies in 2.5- to 3-week-old plants of lettuce genotypes after syringe inoculation. The second leaf was infiltrated with 1 x 10^6^ CFU/mL bacterial inoculum. Leaves were surface sterilized prior to quantification of the bacterial population in the intercellular space. Results are shown as the average of three independent experiments (n = 3 ± standard error). Pairwise mean comparison (bacterial population at day 0 versus day 10) was performed with two-tail Student’s *t*-test (ns, not significant, *p < 0.05, **p < 0.01, and ***p < 0.001).

To assess the genotypic variation in bacterial persistence in the apoplast, a niche where the pathogen is shielded from commonly used sanitation procedures (Erickson, 2012), we directly infiltrated leaves with a needless syringe. This protocol allowed us to exclusively assess the capacity of bacterial persistence in the apoplast of each lettuce genotype, regardless of the bacterial behavior on the phylloplane or differences in leaf surface traits, which might influence bacterial attachment and internalization. Bacterial population kinetics after syringe inoculation differed significantly (p < 0.0001) depending on the bacterium and the lettuce genotype (**Figure 5**). To represent changes on bacterial population dynamics, we calculated the net population growth overtime as fold change (FC) using the ratio 10 DPI/0 DPI (**Figure 5**). Three patterns of bacterial population growth were observed among the genotypes: positive, neutral, and negative growth. *E. coli* O157:H7 exhibited significant positive net growth in seven genotypes ranging from 1.4 ± 0.2 to 2.5 ± 0.1 FC, where the highest growth was observed in the genotypes Red Tide and Bibb (**Figure 5A**). Salinas, Lollo Rossa, and La Brillante showed no significant changes from 0 to 10 DPI and, interestingly, Green Towers showed a net decrease in its population of 0.65 ± 0.05 times (**Figure 5A**). The *S.* Typhimurium 14028s population showed significant positive net growth in only three lettuce genotypes, Red Tide, Serriola I, and Bibb (**Figure 5B**). In contrast, the apoplastic *S.* Typhimurium 14028s population decreased significantly in genotypes Lollo Rossa, Green Towers, and La Brillante by 0.67 ± 0.04, 0.47 ± 0.03, and 0.45 ± 0.03 times, respectively, whereas the *S.* Typhimurium 14028s population did not change significantly in the other five genotypes (**Figure 5B**). Overall, the *E. coli* O157:H7 apoplastic population showed a narrower variation (0.65 < FC < 2.47) inside this panel of lettuce genotypes than that of *S.* Typhimurium 14028s (0.45 < FC < 9.38) (**Figure 5**).

### *S.* Typhimurium 14028s and *E. coli* O157:H7 Persistence in 3.5- to 4-Week-Old Plants After Inoculum Infiltration

**Figure 6:**
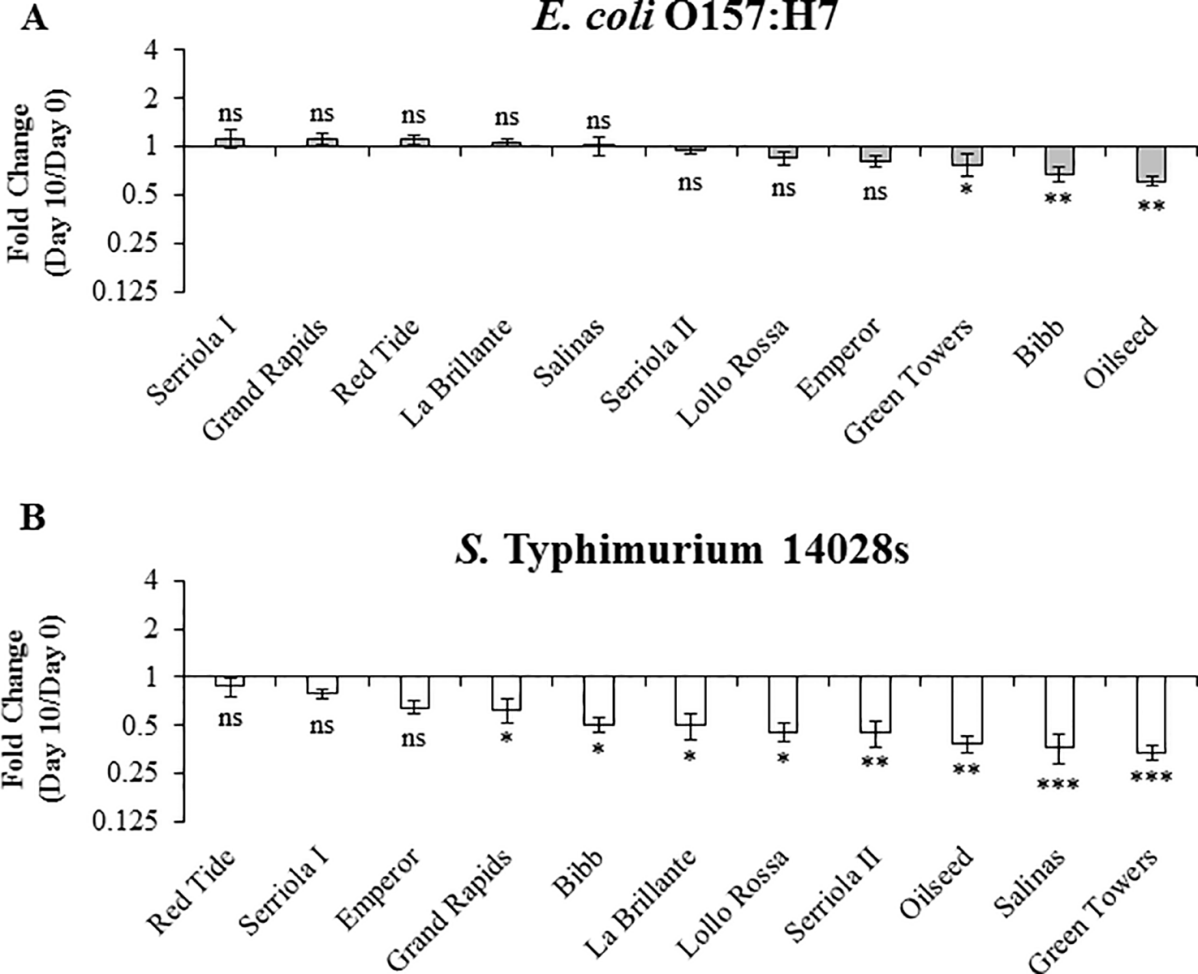
*Escherichia coli* O157:H7 **(A)** and *Salmonella enterica* Typhimurium 14028s **(B)** persistence varies in 3.5- to 4-week-old plants of lettuce genotypes after syringe inoculation. Plants of the lettuce genotypes were grown in pots and then three leaves were infiltrated with bacterial inoculum (1 x 10^6^ CFU/mL) with a needleless syringe. Leaves were surface sterilized after inoculation and serial dilution plating was conducted to quantify the bacterial population in the intercellular space. Three leaves were used for each sample point per genotype. Results are shown as the average of three independent experiments (n = 9 ± standard error). Pairwise mean comparison (bacterial population at day 0 versus day 10) was performed with a two-tail Student’s *t*-test (ns, not significant, *p < 0.05, **p < 0.01, and ***p < 0.001).

**Figure 7:**
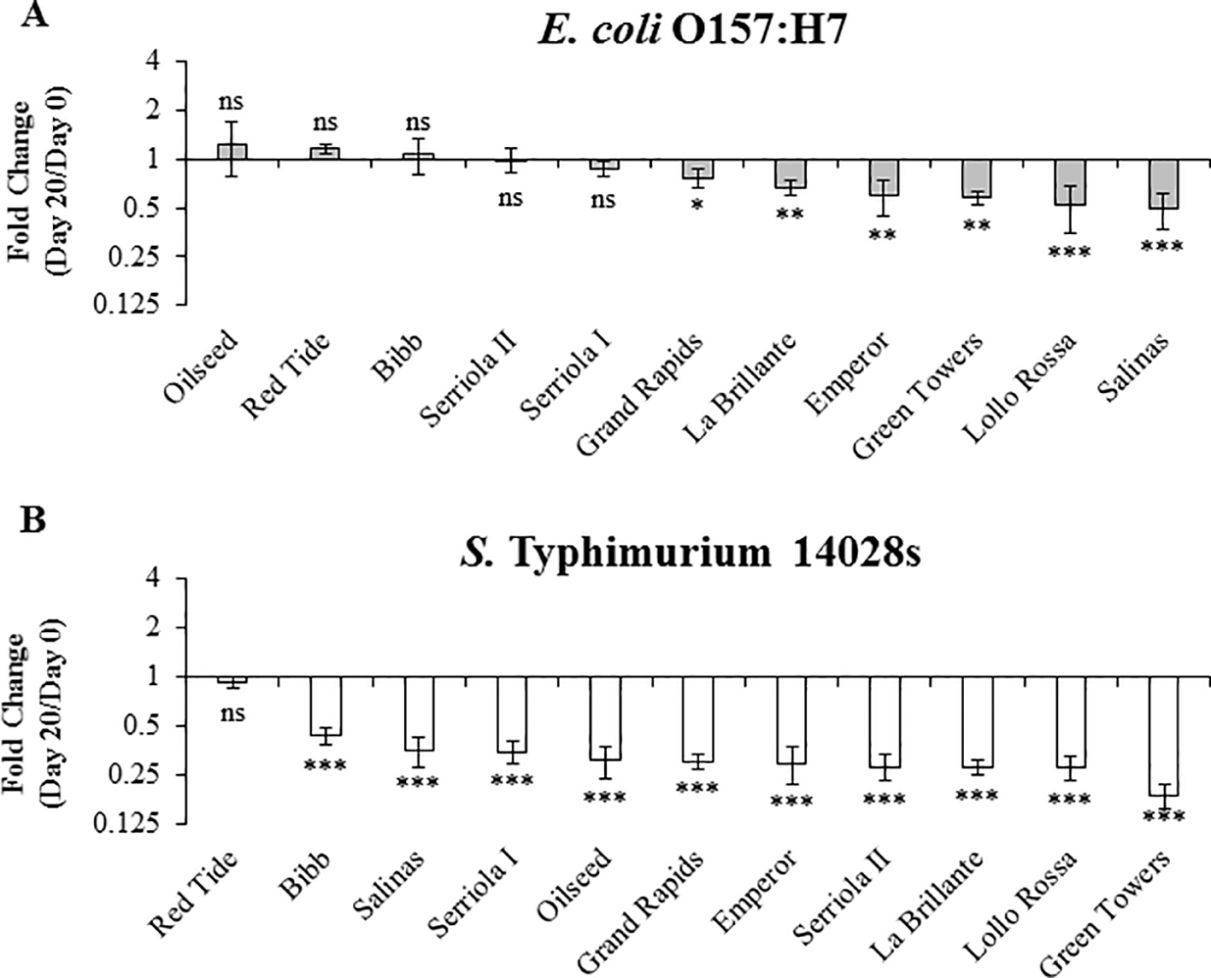
*Escherichia coli* O157:H7 **(A)** and *Salmonella enterica* Typhimurium 14028s **(B)** persistence varies in 3.5- to 4-week-old plants of lettuce genotypes after syringe inoculation. Plants of the lettuce genotypes were grown in pots and then three leaves were infiltrated with bacterial inoculum (1 x 10^6^ CFU/mL) with a needleless syringe. Leaves were surface sterilized after inoculation and serial dilution plating was conducted to quantify the bacterial population in the intercellular space. Three leaves were used for each sample point per genotype. Results are shown as the average of three independent experiments (n = 3 ± standard error). Pairwise mean comparison (bacterial population at day 0 versus day 20) was performed with a two-tail Student’s *t*-test (ns, not significant, *p < 0.05, **p < 0.01, and ***p < 0.001).

Long term survival (≥3 weeks after inoculation) of *E. coli* O157:H7 and *S.* Typhimurium has been previously reported in leaves of species such as Arabidopsis and lettuce Islam et al., 2004a; Islam et al., 2004b; Roy et al., 2013). To explore the effect of plant genetic differences in the survival of human pathogenic bacteria over a longer period of time (i.e., 10 to 20 DPI), we used 3.5- to 4-week-old plants. During this period, no significant positive net population growth was observed for either bacteria in any of the lettuce genotypes (**Figures 6** and **7**). In fact, the population growth of *E. coli* O157:H7 was neutral in five of the lettuce genotypes, Oilseed, Red Tide, Bibb, Serriola II, and Serriola I, during the 20-day period (**Figure 7A**). *E. coli* O157:H7 net growth was negative in the rest of the genotypes, where the smallest FC values were observed in Green Towers, Lollo Rossa, and Salinas (**Figure 7A**). In contrast, Red Tide was the only genotype where *S.* Typhimurium 14028s bacterial population growth remained neutral from 0 to 20 DPI, while the net growth of this bacterium was negative in the other ten genotypes (**Figure 7B**). The genotypes with the smallest FC in the *S.* Typhimurium 14028s population were Lollo Rossa and Green Towers with values of 0.28 ± 0.05 and 0.18 ± 0.03, respectively (**Figure 7B**). Interestingly, the extreme bacterial persistence phenotypes were observed in the same genotypes at the two plant developmental stages (2.5- to 3- and 3.5- to 4-week-old plants) for each human pathogen. In particular, the genotype Red Tide appears to sustain the highest *S.* Typhimurium 14028s and *E. coli* O157:H7 titers, whereas bacterial populations consistently decrease in the genotypes Lollo Rossa, La Brillante, and Green Towers (**Figures 5** and **7**). These differences in apoplastic persistence suggest the existence of constitutive conditions in the apoplast environment and/or induced plant responses that vary among lettuce genotypes and influences bacterial survival.

### *S.* Typhimurium 14028s and *E. coli* O157:H7 ROS Burst Induction in Lettuce Leaves

**Figure 8:**
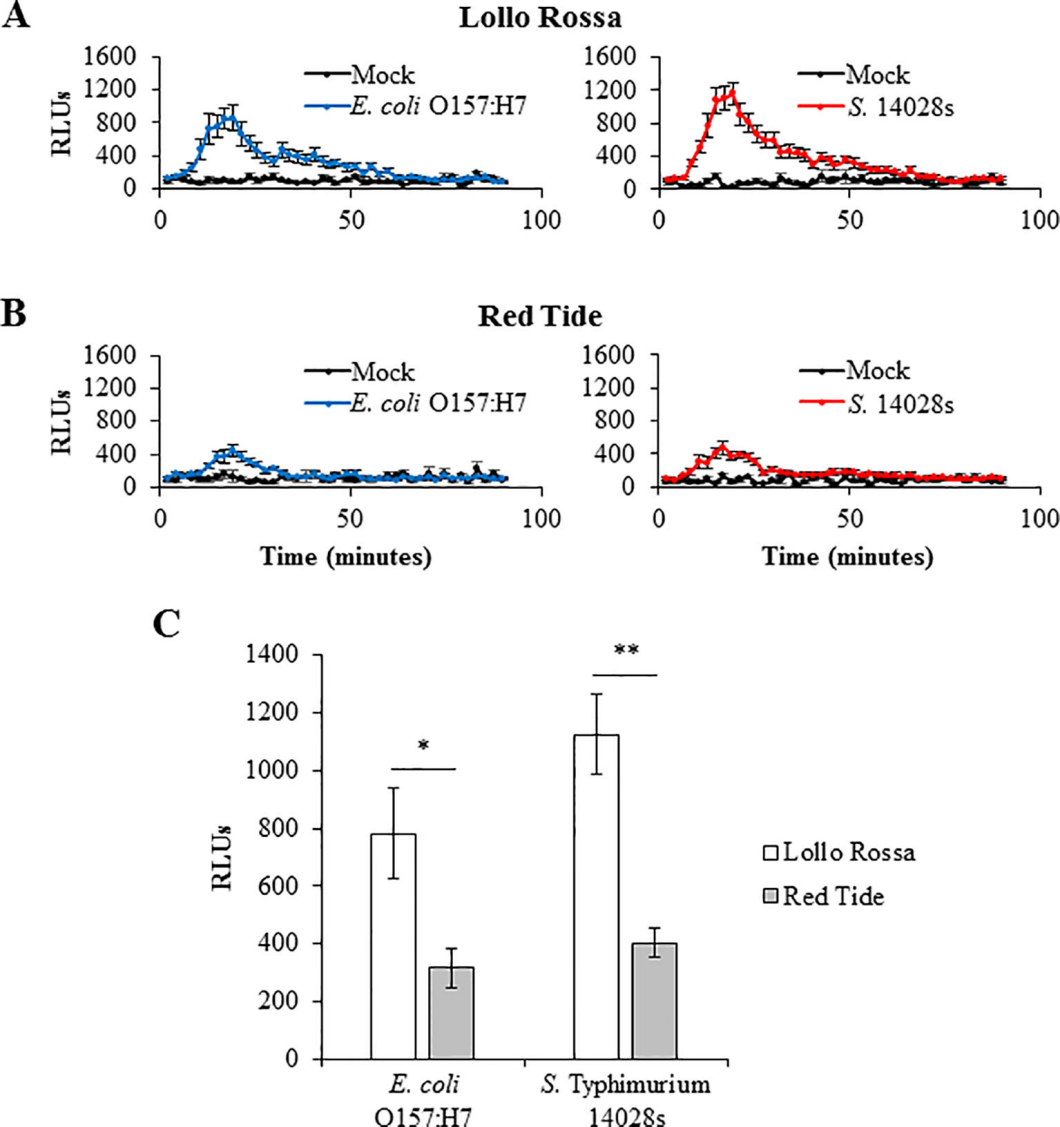
Reactive oxygen species (ROS) burst induced by *Escherichia coli* O157:H7 and *Salmonella enterica* Typhimurium 14028s varies among the lettuce genotypes. The second leaf of Lollo Rossa **(A)** and Red Tide **(B)** plants (2.5- to 3-week-old) was used for the ROS burst assay. Graphs show ROS production after mock- or bacterium-elicitation overtime **(A**, **B)**. ROS production was quantified as relative light units (RLUs). The curve peak (approximately 20 minutes after elicitation) was used to assess statistical significance among the genotypes **(C)**. Results are shown as the RLU peak value (n = 24 ± standard error) of bacterium-treated samples normalized by the mock-treated samples. Data from one out of five independent experiments with similar results are shown. Pairwise mean comparisons (ROS produced by Lollo Rossa versus Red Tide) were performed with a two-tail Student’s *t*-test (*p < 0.05 and **p < 0.001).

To correlate plant defense response levels with bacterial persistence after syringe infiltration inoculation, we chose two lettuce genotypes, Red Tide and Lollo Rossa, which support the two extreme bacterial titers, high and low, respectively (**Figures 5** and **7**), and are parents of a mapping population already characterized by genotyping-by-sequencing. Generation of ROS is among the earliest induced plant cell responses after the perception of MAMP (Yu et al., 2017) and has been reported to occur in Arabidopsis (Garcia et al., 2014) and tobacco (Shirron and Yaron, 2011) after exposure to *S.* Typhimurium 14028s. Consistently, we also observed peak ROS production at approximately 20 minutes after exposure to bacteria; however, the extent of the burst varied significantly between the two plant genotypes after exposure to *E. coli* O157:H7 (p = 0.0117) or *S.* Typhimurium 14028s (p = 0.0001). ROS burst in Lollo Rossa was greater than that of Red Tide after exposure to either *S.* Typhimurium 14028s or *E. coli* O157:H7 (**Figure 8**). The peak of relative light units (RLUs) of Lollo Rossa after treatment with *E. coli* O157:H7 reached 780 ± 156, while Red Tide showed a peak of 315 ± 66 RLUs (**Figure 8C**). After exposure to *S.* Typhimurium 14028s, the peak RLUs in Lollo Rossa was 1,125 ± 140, which was significantly higher than the peak of Red Tide (402 ± 50 RLUs; **Figure 8C**). These results indicate that the differences in bacterial persistence among these genotypes might in part be due to variation in the level of ROS-associated defense responses developed by the plant.

### *S.* Typhimurium 14028s and *E. coli* O157:H7 Differentially Induce Callose Deposition Dependent Upon Lettuce Genotype

**Figure 9:**
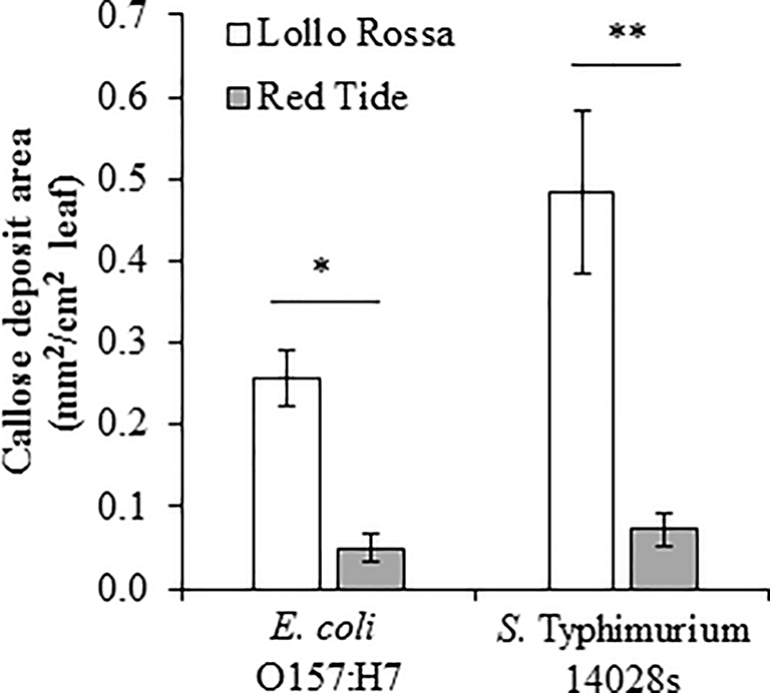
Callose deposition induced by *Escherichia coli* O157:H7 and *Salmonella enterica* Typhimurium 14028s varies among the lettuce genotypes Lollo Rossa and Red Tide. The second leaf of 2.5- to 3-week-old plants was infiltrated with either 1 x 10^8^ CFU/mL bacterial inoculum or water as a control. The area of callose deposition was measured in three plants for each treatment and the experiment was conducted independently four times. Results are shown as mean (n = 12 ± standard error) area of callose deposition normalized with the corresponding mock treatment value. Pairwise mean comparisons (callose deposits produced by Lollo Rossa versus Red Tide) were performed with a two-tail Student’s *t*-test (*< 0.001 and **p < 0.0001).

Another hallmark of plant defense against biotic stressors is callose deposition that occurs within hours after the perception of the microbe (Yu et al., 2017). In lettuce, the induction of callose deposition has been reported as a defense response against phytopathogens such as *Plasmopara lactucae-radicis* (Stanghellini et al., 1993) and *Bremia lactucae* (Cohen et al., 2010). Consistent with the ROS burst response levels, Lollo Rossa exhibited a significantly higher amount of callose deposition than Red Tide after inoculation with either *E. coli* O157:H7 (p = 0.0001) or *S.* Typhimurium 14028s (p < 0.0001; **Figure 9**). Specifically, the average area of callose deposition (mm^2^ callose deposits/cm^2^ leaf) in Lollo Rossa leaves after *E. coli* O157:H7 exposure was 5.2 times greater than Red Tide, and callose deposition after exposure to *S.* Typhimurium 14028s was 6.7 times greater than Red Tide (**Figure 9**). These results suggest that Lollo Rossa is able to generate significantly stronger plant defense responses against *E. coli* O157:H7 and *S.* Typhimurium 14028s than Red Tide, which correlates with the level of bacterial population in the apoplast of these plants.

## Discussion

Despite significant progress achieved in the understanding of the ecology of human pathogens along the food chain environments, there are still relevant unanswered questions regarding molecular mechanisms underlying human pathogen–plant interactions (Barak and Schroeder, 2012; Melotto et al., 2014). The discovery that plant genetic diversity affects the interaction between the phyllosphere and human pathogenic bacteria provided an unprecedented opportunity to dissect the plant components associated with bacterial colonization and persistence phenotypes. However, it is crucial to define significant and robust variables in a genetically tractable system of economic and social importance. Therefore, we conducted a systematic approach to uncover lettuce traits associated with enterobacterium attachment, internalization, and apoplastic persistence.

Lettuce genotypes have extensive variations in leaf traits including the content of surface phenolics, proteins, wax, and sugars; contact angle; and stomatal density (Hunter et al., 2015). These properties have been shown to facilitate or hamper the leaf attachment of human pathogenic bacteria (Golberg et al., 2011; Kroupitski et al., 2011; Hunter et al., 2015). In this study, we observed a significant variation in bacterial attachment among the lettuce genotypes and the differences were dependent upon the bacterial species (**Figure 1**). Our results showed that *E. coli* O157:H7 and *S.* Typhimurium 14028s attachment differs by 1.03 and 0.69 log, respectively, between the extreme genotypes (**Figure 1**). Hunter et al. (2015) reported a 0.22 log difference on leaf attachment of *S. enterica* ser. Senftenberg strain 070885 between *L. sativa* cv. Saladin and *L. serriola* (US96UC23) on the fifth/sixth leaves of 6-week-old plants, but no differences were observed on the first leaf. Furthermore, these authors found a significant correlation between bacterial attachment to leaves and various surface traits, whereas older leaves showed significantly higher levels of bacterial attachment and lower stomatal density than young leaves on the three lettuce genotypes tested (Hunter et al., 2015). Although we found significant differences in the stomatal traits among the lettuce genotypes (**Figure 2**), no significant correlation between these and the attachment level of *E. coli* O157:H7 or *S.* Typhimurium 14028s was detected (**Table 2**). The lack of correlation between these variables might be due to the low number of genotypes used in our study. Nevertheless, Kroupitski et al. (2011) also observed no relationship between differential attachment of *S.* Typhimurium SL1344 on abaxial and adaxial leaf surfaces and the level of stomatal density in romaine lettuce. Overall, the extent of bacterial attachment is the outcome of microbial (Saldaña et al., 2011) and plant (Crawford et al., 2012) physicochemical and biological properties; our results agree with the complex scenario of interactions of each bacterium-lettuce genotype combination.

Successful leaf colonization not only depends on bacterial attachment and epiphytic survival, but also on the ability of the bacteria to penetrate the leaf tissue and occupy the apoplast. Using both microscopy and microbial enumeration tools, several studies have revealed internalization by enteric pathogens through stomata during preharvest plant growth (Erickson, 2012). Leaf penetration of human pathogenic bacteria can be significantly influenced by plant genotype variations (Golberg et al., 2011) and by the tissue site (*i.e.*, different regions of the leaf; Erickson et al., 2010). Using confocal laser scanning microscopy, Golberg et al. (2011) observed significant effects of plant genetic diversity on attachment and internalization of GFP-tagged *S.* Typhimurium, after 2 hours of suspension in bacterial inoculum (1 x 10^8^ CFU/mL). The incidence of internalized bacteria varied among iceberg (81%), romaine (16%), and red (20%) lettuce types (Golberg et al., 2011). Similarly, we have also observed significant variation in the bacterial internalization level among the 11 lettuce genotypes, where the extent of variation between the extreme genotypes was 3.82 times for *E. coli* O157:H7 and 6.08 times for *S.* Typhimurium 14028s (**Figure 3**). Interestingly, taking into consideration the low number of lettuce genotypes used in our study, we were able to detect a significant correlation (p < 0.05) between the IRs of *S.* Typhimurium 14028s or *E. coli* O157:H7 and the stomatal pore traits (**Table 2**). Erickson et al. (2010) suggested that their findings of higher *E. coli* O157:H7 internalization in abaxial versus adaxial-sprayed leaves (1 x 10^8^ CFU/mL inoculum) could be related to higher stomatal density in the leaf abaxial side. Nevertheless, we did not observe a significant correlation between *E. coli* O157:H7 or *S.* Typhimurium 14028s IR and stomatal density (**Table 2**). It is well known that the stomatal immune response also affects the ability of human and plant pathogenic bacteria to internalize leaf tissues (Melotto et al., 2006), and this response is bacterium- and plant-dependent (Roy et al., 2013; Roy and Melotto, 2019). Thus, it is likely that the bacterium internalization of leaves might be influenced not only by certain stomatal morphological traits, such as stomatal aperture width and pore area, but also by stomatal physiological traits and bacterial population dynamics on/in the leaf.

Bacterial access into and survival within the lettuce leaf apoplast pose a risk to consumers and threatens food safety because of the protection that this microenvironment provides to the bacterium from routine sanitization and cleaning treatments (Seo and Frank, 1999; Erickson et al., 2010 ; Golberg et al., 2011; Tomás-Callejas et al., 2011; Ge et al., 2013). Previous studies showing the influence of the plant genotype on bacterial persistence in the phyllosphere considered the total bacterial population (*i.e.*, epiphytic and endophytic populations), as leaves were not surface sterilized prior to bacterium enumeration (Barak et al., 2011; Macarisin et al., 2013). Therefore, the individual contribution of these two distinct niches (*i.e.,* leaf surface and apoplast) to bacterial persistence is not evident. Here, we conducted bacterial persistence assays through surface- and syringe-inoculation methods to distinguish the ability of the bacteria to survive in the leaf apoplast after epiphytic colonization and to persist in the intercellular space regardless of their fitness in the leaf surface, respectively. We found significant differences in the apoplastic persistence of *E. coli* O157:H7 and *S.* Typhimurium 14028s after surface- and syringe-inoculation methods (**Figures 4**–**7**). Although the persistence of inoculated bacteria might have been influenced by a possibly variable sensitivity of each lettuce genotype to the infiltration technique, the inoculation method clearly affected the overall ability of the bacteria to survive in the leaf apoplast of 2.5- to 3-week-old lettuce plants. The capacity of both *E. coli* O157:H7 and *S.* Typhimurium 14028s to persist in the leaf intercellular space was greater after syringe inoculation than after surface inoculation (**Figures 4** and **5**). Lang et al. (2004) reported that significantly higher *E. coli* O157:H7 or *S. enterica* populations were recovered from iceberg lettuce and curly parsley (*Petroselinum crispum*) after dip inoculation compared to spray- or spot-inoculation methods. Higher bacterial adherence after dip inoculation was associated with the detected differences (Lang et al., 2004). Moreover, we observed that bacterial survival during the 10-day experimental period was substantially affected by the inoculation method in certain lettuce genotypes (**Figures 4** and **5**). For example, the lettuce genotype Emperor exhibited the most drastic decline of the *E. coli* O157:H7 population after surface inoculation; however, the *E. coli* O157:H7 population significantly increased after syringe inoculation in this genotype (**Figures 4** and **5**). In contrast, the lettuce genotypes Serriola I, Oilseed, Serriola II, and Bibb presented the highest *E. coli* O157:H7 population titers after both inoculation methods (**Figures 4** and **5**). We hypothesize that the initial epiphytic *S.* Typhimurium 14028s and *E. coli* O157:H7 populations after surface inoculation may either induce plant defense responses and/or be subjected to stress that affect the apoplastic survival and that the extent of these processes might vary according to each bacterium–lettuce genotype combination. For instance, in the case of *E. coli* O157:H7, the stomatal aperture width and pore area showed a significant correlation with the bacterial persistence after surface inoculation (**Table 2**). Therefore, to a certain extent, larger stomatal pores facilitate the leaf penetration of *E. coli* O157:H7 and enhances its apoplastic persistence. This may be due to higher initial internalized bacterial populations. Overall, bacterial persistence after surface inoculation is a complex phenotype, where plant and bacterial factors interact in every step of the colonization.

After syringe inoculations, the net apoplastic growth of *E. coli* O157:H7 and *S.* Typhimurium 14028s was generally higher in 2.5- to 3-week-old plants than in 3.5- to 4-week-old plants at 10 DPI (**Figures 5** and **6**). These results agree with those reported by Brandl and Amundson (2008), where the persistence of *E. coli* O157:H7 and *S. enterica* ser. Thompson strain RM1987 in the romaine lettuce (cultivar Parris Island) phyllosphere was higher in young leaves compared to older leaves, which was associated with the richer total nitrogen and carbon exudates from young leaves. Moreover, the relative bacterial persistence after syringe inoculation between the lettuce genotypes was slightly affected by the developmental stage of the lettuce plants (**Figures 5**–**7**). For instance, *E. coli* O157:H7 and *S.* Typhimurium 14028s exhibited the highest levels of apoplastic survival in the genotype Red Tide after syringe inoculation of 2.5- to 3-week-old plants (**Figure 5**) and 3.5- to 4-week-old plants (**Figures 6** and **7**). Likewise, the lowest levels of bacterial persistence in the leaves of Green Towers were observed in plants of the two developmental stages used in our study (**Figures 5**–**7**). Thus, factors determining bacterial apoplastic survival after syringe inoculation might change proportionally over time depending on the lettuce genotypes.

It has been previously shown that plant immune responses may be activated by human pathogens (Barak and Schroeder, 2012; Roy et al., 2013; Melotto et al., 2014; Jo and Park, 2019). However, plant immune responses have not been correlated with the level of *E. coli* O157:H7 and *S.* Typhimurium 14028s population growth. Therefore, we proceeded to assess hallmark PTI responses (Yu et al., 2017) with the lettuce genotypes Lollo Rossa and Red Tide that showed contrasting phenotypes in bacterial persistence after bacterial infiltration. The lettuce genotype Lollo Rossa, which exhibited one of the lowest bacterial apoplastic persistence levels after syringe inoculation, showed a significantly higher ROS burst and callose deposition than Red Tide (**Figures 8** and **9**). ROS burst and callose deposition were also reported to be generated by Arabidopsis plants after exposure to 1 μM of the flagellin epitope flg22 of *S.* Typhimurium 14028s (Garcia et al., 2014). Additionally, in lettuce, the induction of callose deposition has been observed as a defense response against lettuce phytopathogens (Stanghellini et al., 1993; Cohen et al., 2010), while oxidative stress has been associated with the non-host hypersensitive reaction against the bacteria *Pseudomonas syringae* pv. *phaseolicola* (Bestwick et al., 1998; Bestwick et al., 2001). These results strongly suggest that the differences in the ability of *E. coli* O157:H7 and *S.* Typhimurium 14028s to survive in the leaf apoplast of the genotypes Lollo Rossa and Red Tide are influenced by the variation in the level of defense responses activated against these bacteria. Although the contribution of the type III secretion systems and type III effectors in the colonization of plants by human pathogenic bacteria remains controversial (Schikora et al., 2011; Shirron and Yaron, 2011; Melotto et al., 2014; Chalupowicz et al., 2018; Montano et al., 2020), it is possible that the lettuce genotypes differ in their ability to recognize type III effectors of *S.* Typhimurium 14028s and *E. coli* O157:H7 resulting in variation in effector-triggered immunity against these human pathogenic bacteria. Interestingly, Lollo Rossa has been reported as resistant to the disease bacterial leaf spot of lettuce caused by *Xanthomonas campestris* pv. *vitians* (**Table 1**; Hayes et al., 2014), which suggests that this lettuce genotype might have a strong basal immune system to a wide range of bacteria. In tomato leaves, Potnis et al. (2015) observed that induction of water-soaked lesions by *X. euvesicatoria* or *X. gardneri* (causal agents of tomato bacterial spot disease) promoted *S. enterica* (serovars Enteritidis and Baildon cocktail) growth. Taking this into consideration, it is possible that the *X. campestris* pv. *vitians* interaction with lettuce could also provide a conducive environment for the growth of *Salmonella*. Therefore, Lollo Rossa could potentially possess traits that might contribute directly and indirectly to the prevention of human pathogenic bacterial survival in the phyllosphere. In addition, it has been shown that the composition of natural microbiota in the lettuce phyllosphere can be significantly influenced by leaf properties (Hunter et al., 2010) and affect the leaf colonization by human pathogenic bacteria (Lima et al., 2013). Possibly, differences in indigenous microbial communities among the lettuce genotypes used in our study might have contributed to the observed variation on *E. coli* O157:H7 and *S.* Typhimurium 14028s leaf colonization. The apoplastic survival of *E. coli* O157:H7 after syringe inoculation was in general higher than the apoplastic survival of *S.* Typhimurium 14028s in Lollo Rossa and the other lettuce genotypes (**Figures 5**–**7**). This agrees with the overall stronger immune responses of Lollo Rossa to *S.* Typhimurium 14028s compared to *E. coli* O157:H7 (**Figures 8** and **9**). This variation resembles quantitative resistance, where the phenotype is polygenically controlled and the predominant mechanisms extend beyond differences in pathogen recognition to variation in defense-related outputs such as strengthening of the cell wall or defense compound biosynthesis (Corwin and Kliebenstein, 2017). Determining the genetic bases of this phenotype is key for the potential incorporation into lettuce breeding programs towards enhanced food safety.

## Data Availability Statement

The datasets generated for this study are available upon request to the corresponding author.

## Author Contributions

MM conceived research. CJ performed the experiments. CJ and MM designed the research, analyzed the data, and wrote the manuscript. All authors read and approved the manuscript.

## Funding

This research was supported by grants from the U.S. Department of Agriculture – National Institute of Food and Agriculture (NIFA; 2015-67017-23360 and 2017-67017-26180) and NIFA Hatch grant (CA-D-PLS-2327-H) to MM. CJ was supported by a BECAS-Chile (CONICYT) fellowship and Horticulture and Agronomy Graduate Group fellowship from the University of California, Davis.

## Conflict of Interest

The authors declare that the research was conducted in the absence of any commercial or financial relationships that could be construed as a potential conflict of interest.

## References

[B1] Allende A. Monaghan J. (2015). Irrigation water quality for leafy crops: a perspective of risks and potential solutions. Int. J. Environ. Res. Public Health 12, 7457–7477. 10.3390/ijerph120707457 26151764 PMC4515668

[B2] Barak J. D. Schroeder B. K. (2012). Interrelationships of food safety and plant pathology: the life cycle of human pathogens on plants. Annu. Rev. Phytopathol. 50, 241–266. 10.1146/annurev-phyto-081211-172936 22656644

[B3] Barak J. D. Kramer L. C. Hao L. (2011). Colonization of tomato plants by *Salmonella enterica* is cultivar dependent, and type 1 trichomes are preferred colonization sites. Appl. Environ. Microbiol. 77, 498–504. 10.1128/AEM.01661-10 21075871 PMC3020540

[B4] Bennett S. D. Sodha S. V. Ayers T. L. Lynch M. F. Gould L. H. Tauxe R. V. (2018). Produce-associated foodborne disease outbreaks, USA, 1998–2013. Epidemiol. Infect. 11, 1397–1406. 10.1017/S0950268818001620 PMC9133681 29923474

[B5] Bestwick C. S. Brown I. R. Mansfield J. W. (1998). Localized changes in peroxidase activity accompany hydrogen peroxide generation during the development of a nonhost hypersensitive reaction in lettuce. Plant Physiol. 118, 1067–1078. 10.1104/pp.118.3.1067 9808752 PMC34780

[B6] Bestwick C. S. Adam A. L. Puri N. Mansfield J. W. (2001). Characterisation of and changes to pro- and anti-oxidant enzyme activities during the hypersensitive reaction in lettuce (*Lactuca sativa* L.). Plant Sci. 161, 497–506. 10.1016/S0168-9452(01)00427-7

[B7] Brandl M. T. Amundson R. (2008). Leaf age as a risk factor in contamination of lettuce with *Escherichia coli* O157:H7 and *Salmonella enterica* . Appl. Environ. Microbiol. 74, 2298–2306. 10.1128/AEM.02459-07 18310433 PMC2293143

[B8] Chalupowicz L. Nissan G. Brandl M. T. McClelland M. Sessa G. Popov,. G. (2018). Assessing the ability of *Salmonella enterica* to translocate type III effectors into plant cells. Mol. Plant Microbe In. 31, 233–239. 10.1094/MPMI-07-17-0166-R 28952399

[B9] Cohen Y. Rubin A. E. Kilfin G. (2010). Mechanisms of induced resistance in lettuce against *Bremia lactucae* by DL-β-amino-butyric acid (BABA). Eur. J. Plant Pathol. 126, 553–573. 10.1007/s10658-009-9564-6

[B10] Cook R. (2016). Fresh-cut/value-added produce marketing trends. UC Davis fresh-cut products workshop: maintaining quality and safety (Davis, CA, USA). Retrieved April, 11, 2019 from: https://arefiles.ucdavis.edu/uploads/filer_public/fb/7b/fb7b6380-cdf9-4db5-b5d2-993640bcc1e6/freshcut2016cook20160926final.pdf.

[B11] Corwin J. A. Kliebenstein D. J. (2017). Quantitative resistance: more than just perception of a pathogen. Plant Cell 29, 655–665. 10.1105/tpc.16.00915 28302676 PMC5435431

[B12] Crawford R. J. Webb H. K. Truong V. K. Hasan J. Ivanova E. P. (2012). Surface topographical factors influencing bacterial attachment. Adv. Colloid Interface Sci. 179–182, 142–149. 10.1016/j.cis.2012.06.015 22841530

[B13] Crozier L. Hedley P. E. Morris J. Wagstaff C. Andrews S. C. Toth I. (2016). Whole-Transcriptome analysis of verocytotoxigenic *Escherichia coli* O157:H7 (Sakai) suggests plant-species-specific metabolic responses on exposure to spinach and lettuce extracts. Front. Microbiol. 7, 1088. 10.3389/fmicb.2016.01088 27462311 PMC4940412

[B14] Erickson M. C. Webb C. C. Diaz-Perez J. C. Phatak S. C. Silvoy J. J. Davey,. L. (2010). Surface and internalized *Escherichia coli* O157:H7 on field-grown spinach and lettuce treated with spray-contaminated irrigation water. J. Food Prot. 73, 1023–1029. 10.1146/annurev-food-022811-101211 20537256

[B15] Erickson M. C. Liao J. Y. Payton A. S. Cook P. W. Den Bakker H. C. Bautista J. (2018). Fate of enteric pathogens in different spinach cultivars cultivated in growth chamber and field systems. Food Quality Safety 2, 221–228. 10.1093/fqsafe/fyy020

[B16] Erickson M. C. (2012). Internalization of fresh produce by foodborne pathogens. Annu. Rev. Food Sci. Technol. 3, 283–310. 10.1146/annurev-food-022811-101211 22243280

[B17] ERS-USDA (Economic Research Service - United States Department of Agriculture) (2018). Vegetables and pulses yearbook data. U.S. per capita use of fresh and processing vegetables, dry pulse crops, and potatoes; cash receipts; U.S. vegetable trade. Retrieved April, 11, 2019 from: https://www.ers.usda.gov/data-products/vegetables-and-pulses-data/vegetables-and-pulses-yearbook-tables/.

[B18] Fonseca J. M. Fallon S. D. Sanchez C. A. Nolte K. D. (2011). *Escherichia coli* survival in lettuce fields following its introduction through different irrigation systems. J. Appl. Microbiol. 110, 893–902. 10.1111/j.1365-2672.2011.04942.x 21214696

[B19] Garcia A. V. Charrier A. Schikora A. Bigeardm J. Pateyron S. de Tauzia-Moreau M. L. (2014). *Salmonella enterica* flagellin is recognized *via* FLS2 and activates PAMP-Triggered immunity in *Arabidopsis thaliana* . Mol. Plant 7, 657–674. 10.1093/mp/sst145 24198231

[B20] Ge C. Bohrerova Z. Lee J. (2013). Inactivation of internalized *Salmonella* Typhimurium in lettuce and green onion using ultraviolet C irradiation and chemical sanitizers. J. Appl. Microbiol. 114, 1415–1424. 10.1111/jam.12154 23351161

[B21] Golberg D. Kroupitski Y. Belausov E. Pinto R. Sela S. (2011). *Salmonella* Typhimurium internalization is variable in leafy vegetables and fresh herbs. Int. J. Food Microbiol. 145, 250–257. 10.1016/j.ijfoodmicro.2010.12.031 21262550

[B22] Grube R. C. Ochoa O. E. (2005). Comparative genetic analysis of field resistance to downy mildew in the lettuce cultivars ‘Grand Rapids’ and ‘Iceberg’. Euphytica 142, 205–215. 10.1007/s10681-005-1683-3

[B23] Grube R. C. Ryder E. J. Koike S. T. McCreight J. D. Wintermantel W. M. (2003). “Breeding for resistance to new and emerging lettuce diseases in California,” in Eucarpia Leafy Vegetables 2003. Eds. Th van Hintum J. L. Lebeda A. Pink D. Schut J. W., 37–42. ^©^ CGN Wageningen, Netherlands.

[B24] Gutiérrez-Rodríguez E. Gundersen A. Sbodio A. O. Suslow T. V. (2011). Variable agronomic practices, cultivar, strain source and initial contamination dose differentially affect survival of *Escherichia coli* on spinach. J. Appl. Microbiol. 112, 109–118. 10.1111/j.1365-2672.2011.05184.x 22040351

[B25] Han S. Micallef S. A. (2014). *Salmonella* Newport and Typhimurium colonization of fruit differs from leaves in various tomato cultivars. J. Food Prot. 77, 1844–185. 10.4315/0362-028X.JFP-13-562 25364916

[B26] Hayes R. J. Wu B. M. Pryor B. M. Chitrampalam P. Subbarao K. V. (2010). Assessment of resistance in lettuce (*Lactuca sativa* l.) to mycelial and ascospore infection by *Sclerotinia minor* Jagger and *S. sclerotiorum* (Lib.) de Bary. HortScience 45, 333–341. 10.21273/HORTSCI.45.3.333

[B27] Hayes R. J. Trent M. A. Mou B. Simko I. Gebben S. J. Bull C. T. (2014). Baby leaf lettuce germplasm enhancement: developing diverse populations with resistance to bacterial leaf spot caused by *Xanthomonas campestris* pv. *vitians* . HortScience 49, 18–24. 10.21273/HORTSCI.49.1.18

[B28] Hunter P. J. Hand P. Pink D. Whipps J. M. Bending G. D. (2010). Both leaf properties and microbe-microbe interactions influence within-species variation in bacterial population diversity and structure in the lettuce (*Lactuca* Species) phyllosphere. Appl. Environ. Microbiol. 76, 8117–8125. 10.1128/AEM.01321-10 20952648 PMC3008232

[B29] Hunter P. J. Shaw R. K. Berger C. N. Frankel G. Pink D. Hand P. (2015). Older leaves of lettuce (*Lactuca* spp.) support higher levels of *Salmonella enterica* ser. Senftenberg attachment and show greater variation between plant accessions than do younger leaves. FEMS Microbiol. Lett. 362, fnv077. 10.1093/femsle/fnv077 25953858 PMC7613271

[B30] Islam M. Morgan J. Doyle M. P. Phatak S. C. Millner P. Jiang X. (2004a). Persistence of *Salmonella enterica* Serovar Typhimurium on lettuce and parsley and in soils on which they were grown in fields treated with contaminated manure composts or irrigation water. Foodborne Pathog. Dis. 1, 27–35. 10.1089/153531404772914437 15992259

[B31] Islam M. Doyle M. P. Phatak S. C. Millner P. Jiang X. (2004b). Persistence of enterohemorrhagic *Escherichia coli* O157:H7 in soil and on leaf lettuce and parsley grown in fields treated with contaminated manure composts or irrigation water. J. Food Prot. 67, 1365–1370. 10.4315/0362-028x-67.7.1365 15270487

[B32] Jacob C. Panchal S. Melotto M. (2017). Surface inoculation and quantification of *Pseudomonas syringae* population in the Arabidopsis leaf apoplast. Bio-protocol 7 (5), e2167. 10.21769/BioProtoc.2167 28573169 PMC5448416

[B33] Jay-Russell M. T. (2013). What is the risk from wild animals in food-borne pathogen contamination of plants? CAB Reviews: Perspectives Agriculture Veterinary Science Nutrition Natural Resources 8, 1–6. 10.1079/PAVSNNR20138040

[B34] Jiang X. Chen Z. Dharmasena M. (2015). “The role of animal manure in the contamination of fresh food, Chapter 13, pp 312-350,” in Advances in microbial food safety, Volume 2. Ed. Sofos J. (Oxford, UK: Woodhead Publishing-Elsevier Limited), 405.

[B35] Jo S. H. Park J. M. (2019). The dark side of organic vegetables: interactions of human enteropathogenic bacteria with plants. Plant Biotechnology Reports 13, 105–110. 10.1007/s11816-019-00536-1

[B36] Katagiri F. Thilmony R. He S. Y. (2002). The *Arabidopsis thaliana*-*Pseudomonas syringae* interaction. Arabidopsis Book. 1, e0039. 10.1199/tab.0039 22303207 PMC3243347

[B37] Kisluk G. Yaron S. (2012). Presence and persistence of *Salmonella enterica* serotype Typhimurium in the phyllosphere and rhizosphere of spray-irrigated parsley. Appl. Environ. Microbiol. 78, 4030–4036. 10.1128/AEM.00087-12 22447598 PMC3346414

[B38] Klerks M. M. Franz E. van Gent-Pelzer M. Zijlstra C. van Bruggen A. H. C. (2007). Differential interaction of *Salmonella enterica* serovars with lettuce cultivars and plant-microbe factors influencing the colonization efficiency. ISME J. 1, 620–631. 10.1038/ismej.2007.82 18043669

[B39] Kroupitski Y. Golberg D. Belausov E. Pinto R. Swartzberg D. Granot D. (2009). Internalization of *Salmonella enterica* in leaves is induced by light and involves chemotaxis and penetration through open stomata. Appl. Environ. Microbiol. 75, 6076–6086. 10.1128/AEM.01084-09 19648358 PMC2753090

[B40] Kroupitski Y. Pinto R. Belausov E. Sela S. (2011). Distribution of *Salmonella typhimurium* in romaine lettuce leaves. Food Microbiol. 28, 990–997. 10.1016/j.fm.2011.01.007 21569943

[B41] Lang M. M. Harris L. J. Beuchat L. R. (2004). Survival and recovery of *Escherichia coli* O157:H7, *Salmonella*, and *Listeria monocytogenes* on lettuce and parsley as affected by method of inoculation, time between inoculation and analysis, and treatment with chlorinated water. J. Food Prot. 67, 1092–1103. 10.4315/0362-028X-67.6.1092 15222533

[B42] Lima P. M. São José J. F. B. Andrade N. J. Pires A. C. S. Ferreira S. O. (2013). Interaction between natural microbiota and physicochemical characteristics of lettuce surfaces can influence the attachment of *Salmonella* Enteritidis. Food Control 30, 157–161. 10.1016/j.foodcont.2012.06.039

[B43] Macarisin D. Patel J. Bauchan G. Giron J. A. Ravishankar S. (2013). Effect of spinach cultivar and bacterial adherence factors on survival of *Escherichia coli* O157:H7 on spinach leaves. J. Food Prot. 76, 1829–1837. 10.4315/0362-028X.JFP-12-556 24215684

[B44] Manikandan S. (2010). Data transformation. J. Pharmacol. Pharmacother. 1, 126–127. 10.4103/0976-500X.72373 21350629 PMC3043340

[B45] Marvasi M. Noel T. George A. S. Farias M. A. Jenkins K. T. Hochmuth G. (2014). Ethylene signaling affects susceptibility of tomatoes to *Salmonella* . Microb. Biotechnol. 7, 545–555. 10.1111/1751-7915.12130 24888884 PMC4265073

[B46] McCreight J. D. Matheron M. E. Tickes B. R. Platts B. (2005). Fusarium wilt race 1 on lettuce. HortScience 40, 529–531. 10.21273/HORTSCI.40.3.529

[B47] Melotto M. Underwood W. Koczan J. Nomura K. He S. Y. (2006). Plant stomata function in innate immunity against bacterial invasion. Cell 126, 969–980. 10.1016/j.cell.2006.06.05 16959575

[B48] Melotto M. Panchal S. Roy D. (2014). Plant innate immunity against human bacterial pathogens. Front. Microbiol. 5, 1–12. 10.3389/fmicb.2014.00411 25157245 PMC4127659

[B49] Mitra R. Cuesta-Alonso E. Wayadande A. Talley J. Gilliland S. Fletcher J. (2009). Effect of route of introduction and host cultivar on the colonization, internalization, and movement of the human pathogen *Escherichia coli* O157:H7 in spinach. J. Food Prot. 72, 1521–1530. 10.4315/0362-028x-72.7.1521 19681281

[B50] Montano J. Rossidivito G. Torreano J. Porwollik S. Sela S. McClelland M. (2020). *Salmonella enterica* serovar Typhimurium 14028s genomic regions required for colonization of lettuce leaves. Front. Microbiol. (in press). 10.3389/fmicb.2020.00006 PMC6993584 32038592

[B51] Porwollik S. Santiviago S. A. Cheng P. Long F. Desai P. Fredlund J. (2014). Defined single-gene and multi-gene deletion mutant collections in *Salmonella enterica* sv Typhimurium. PLoS ONE 9, e99820. 10.1371/journal.pone.0099820 25007190 PMC4089911

[B52] Potnis N. Colee J. Jones J. B. Barak J. D. (2015). Plant pathogen-induced water-soaking promotes *Salmonella enterica* growth on tomato leaves. Appl. Environ. Microbiol. 81, 8126–8134. 10.1128/AEM.01926-15 26386057 PMC4651078

[B53] Quilliam R. S. Williams A. P. Jones D. L. (2012). Lettuce cultivar mediates both phyllosphere and rhizosphere activity of *Escherichia coli* O157:H7. PLoS ONE 7, e33842. 10.1371/journal.pone.0033842 22439006 PMC3306295

[B54] Roy D. Melotto M. (2019). Stomatal response and human pathogen persistence in leafy greens under preharvest and postharvest environmental conditions. Postharvest Biol. Technol. 148, 76–82. 10.1016/j.postharvbio.2018.10.013

[B55] Roy D. Panchal S. Rosa B. A. Melotto M. (2013). *Escherichia coli* O157:H7 induces stronger plant immunity than *Salmonella enterica* Typhimurium SL1344. Phytopathology 103, 326–332. 10.1094/PHYTO-09-12-0230-FI 23301812 PMC3982233

[B56] Ryder E. J. Robinson B. J. (1995). Big-vein resistance in lettuce: identifying, selecting, and testing resistant cultivars and breeding lines. J. Amer. Soc. Hort. Sci. 120, 741–746. 10.21273/JASHS.120.5.741

[B57] Saldaña Z. Sánchez E. Xicohtencatl-Cortes J. Puente J. L. Girón J. A. (2011). Surface structures involved in plant stomata and leaf colonization by Shiga-toxigenic *Escherichia coli* O157:H7. Front. Microbiol. 27, 119. 10.3389/fmicb.2011.00119 PMC3157101 21887151

[B58] Sapers G. M. Doyle M. P. (2014). “Chapter 1 - Scope of the produce contamination problem. p. 3-20,” in The produce contamination problem: causes and solutions. Second Edition. Eds. Matthews K. R. Sapers G. M. Gerba C. P. (Amsterdam, Netherlands: Elsevier), 467.

[B59] Schenk S. T. Schikora A. (2015). Staining of callose depositions in root and leaf tissues. Bio-protocol 5, e1429. 10.21769/BioProtoc.1429

[B60] Schikora A. Virlogeux-Payant I. Bueso E. Garcia A. V. Nilau T. Charrieret A. (2011). Conservation of *Salmonella* infection mechanisms in plants and animals. PLoS ONE 6, e24112. 10.1371/journal.pone.0024 21915285 PMC3167816

[B61] Scott J. C. Kirkpatrick S. C. Gordon T. R. (2010). Variation in susceptibility of lettuce cultivars to fusarium wilt caused by *Fusarium oxysporum* f.sp. *lactucae* . Plant Pathol. 59, 139–146. 10.1111/j.1365-3059.2009.02179.x

[B62] Seo K. H. Frank J. F. (1999). Attachment of *Escherichia coli* O157:H7 to lettuce leaf surface and bacterial viability in response to chlorine treatment as demonstrated by using confocal scanning laser microscopy. J. Food Prot. 62, 3–9. 10.4315/0362-028x-62.1.3 9921820

[B63] Shirron N. Yaron S. (2011). Active suppression of early immune response in tobacco by the human pathogen *Salmonella* Typhimurium. PLoS ONE 6, e18855. 10.1371/journal.pone.0018855 21541320 PMC3082535

[B64] Simko I. Pechenick D. A. McHale L. K. Truco M. J. Ochoa O. E. Michelmore R. W. (2009). Association mapping and marker-assisted selection of the lettuce dieback resistance gene Tvr1. BMC Plant Biol. 9, 135. 10.1186/1471-2229-9-135 19930659 PMC2789080

[B65] Simko I. Rauscher G. Sideman R. G. McCreight J. D. Hayes R. J. (2014). Evaluation and QTL mapping of resistance to powdery mildew in lettuce. Plant Pathol. 63, 344–353. 10.1111/ppa.12087

[B66] Simko I. Ochoa O. E. Pel M. A. Tsuchida C. Font C. Forcada I. (2015). Resistance to downy mildew in lettuce ‘La Brillante’ is conferred by Dm50 gene and multiple QTL. Phytopathology 105, 1220–1228. 10.1094/PHYTO-02-15-0057-R 25915441

[B67] Smith J. M. Heese A. (2014). Rapid bioassay to measure early reactive oxygen species production in Arabidopsis leave tissue in response to living *Pseudomonas syringae* . Plant Methods 10, 6. 10.1186/1746-4811-10-6 24571722 PMC3941562

[B68] Solomon E. B. Pang H. J. Matthews K. R. (2003). Persistence of *Escherichia coli* O157:H7 on lettuce plants following spray irrigation with contaminated water. J. Food Prot. 66, 2198–2202. 10.4315/0362-028X-66.12.2198 14672213

[B69] Sperandio V. Torres A. G. Girón J. A. Kaper J. B. (2001). Quorum sensing is a global regulatory mechanism in entrohemorrhagic *Escherichia coli* O157:H7. J. Bac. 183, 5187–5197. 10.1128/JB.183.17.5187-5197.2001 PMC95396 11489873

[B70] Stanghellini M. E. Rasmussen S. L. Vandemark G. J. (1993). Relationship of callose deposition to resistance of lettuce to *Plasmopara lactucae-radicis* . Phytopathology 83, 1498–1501. 10.1094/Phyto-83-1498

[B71] Tomás-Callejas A. López-Velasco G. Camacho A. B. Artés F. Artés-Hernández F. Suslow T. V. (2011). Survival and distribution of *Escherichia coli* on diverse fresh-cut baby leafy greens under preharvest through postharvest conditions. Int. J. Food Microbiol. 151, 216–222. 10.1016/j.ijfoodmicro.2011.08.027 21924789

[B72] Vallad G. E. Subbarao K. V. (2008). Colonization of resistant and susceptible lettuce cultivars by a green fluorescent protein-tagged isolate of *Verticillium dahliae* . Phytopathology 98, 871–885. 10.1094/PHYTO-98-8-0871 18943205

[B73] Van der Linden I. Cottyn B. Uyttendaele M. Vlaemynck G. Maes M. Heyndrickx M. (2014). Evaluation of an attachment assay on lettuce leaves with temperature- and starvation-stressed *Escherichia coli* O157:H7 MB3885. J. Food Prot. 77, 549–557. 10.4315/0362-028X.JFP-13-332 24680065

[B74] Van der Linden I. Cottyn B. Uyttendaele M. Vlaemynck G. Heyndrickx M. Maes M. (2016). Microarray-based screening of differentially expressed genes of *E. coli* O157:H7 Sakai during preharvest survival on butterhead lettuce. Agriculture 6, 6. 10.3390/agriculture6010006

[B75] Whipps J. M. Budge S. P. McClement S. Pink D. A. C. (2002). A glasshouse cropping method for screening lettuce lines for resistance to *Sclerotinia sclerotiorum* . Eur. J. Plant Pathol. 108, 373–378. 10.1023/A1015637018474

[B76] Wong C. W. Y. Wang S. Lévesque R. C. Goodridge L. Delaquis P. (2019). Fate of 43 *Salmonella* strains on lettuce and tomato seedlings. J. Food Prot. 82, 1045–1051. 10.4315/0362-028X.JFP-18-435 31124714

[B77] Yaron S. Römling U. (2014). Biofilm formation by enteric pathogens and its role in plant colonization and persistence. Microb. Biotechnol. 7, 496–516. 10.1111/1751-7915.12186 25351039 PMC4265070

[B78] Yu X. Feng B. He P. Shan L. (2017). From chaos to harmony: responses and signaling upon microbial pattern recognition. Annu. Rev. Phytopathol. 55, 109–137. 10.1146/annurev-phyto-080516-035649 28525309 PMC6240913

